# Snakeleev: A Gamified Serious Game for Learning the Periodic Table

**DOI:** 10.1021/acs.jchemed.5c00029

**Published:** 2025-04-29

**Authors:** Pietro Galizia

**Affiliations:** National Research Council of Italy - Institute of Science, Technology and Sustainability for Ceramics (CNR - ISSMC), Via Granarolo 64, 48018 Faenza, Italy

**Keywords:** (Audience): General Public, High School/Introductory Chemistry, Continuing Education. (Domain): Interdisciplinary/Multidisciplinary, Public Understanding/Outreach. (Pedagogy): Distance Learning/Self Instruction, Humor/Puzzles/Games, Internet/Web-Based Learning, Mnemonics/Rote Learning. (Topic): Atomic Properties/Structure, Periodicity/Periodic Table

## Abstract

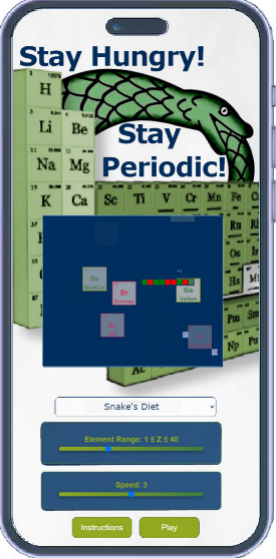

This work introduces *Snakeleev*, a gamified serious game designed to enhance learning and memorization of the periodic table of chemical elements through an engaging and interactive experience (https://pietrogalizia.github.io/Snakeleev/). For the first time, *Snakeleev* transforms the classic Snake game into an educational tool, replacing apples with chemical elements and challenging players to recognize and classify them based on thematic “diets” tied to real-world applications, such as smartphone components and critical raw materials. By integrating gamification, active learning, and real-world applications, this Snake-based video game fosters interdisciplinary connections, linking the periodic table to materials science, sustainability, and technology. Its thematic diets range from energy and electronics to biology, geology, and astronomy, while some explore socioeconomic and linguistic aspects, such as Latin and Greek etymologies. A preliminary statistical analysis suggested a positive learning effect. After 10 and 20 min of gameplay, students’ scores improved markedly, with Cohen’s d ranging from 1.23 (indicating very large effect for symbol-to-name association of chemical elements) to 2.67 (huge effect for classification by diet). The greatest learning gains occurred within the first 10 min, particularly on less familiar topics. The Friedman test (*p* < 0.0001) indicated statistically significant improvements. Surveys showed that over 90% of students found *Snakeleev* engaging and helpful, with many recommending it, stating: “It definitely saves me hours of studying” and “Even without trying, you automatically learn the periodic table quickly”. Beyond chemistry, *Snakeleev*’s innovative and pioneering framework enables future expansions into other disciplines, including *Snakileo Snakilei* (physics), *Phytonacci* (mathematics), and *SnEco* (waste sorting), extending its application not only to science education but also to other fields. By merging education and entertainment, *Snakeleev* stands as the first and original gamified Snake-based serious game, reimagining *Snake* not only to explore the periodic table of elements, but also to foster curiosity and interdisciplinary exploration across STEM, humanities, and environmental education.

## Introduction

*“Chemistry is about the chemical elements”*, which can be ordered in a periodic table.^1^ As fundamental pillar in chemistry education, the periodic table provides a structured framework for understanding the electron configurations of elements, their chemical behaviors, and the properties of the substances they form.^2−6^ However, traditional teaching methods often rely on rote memorization techniques, which can disengage students and hinder deeper comprehension of the periodic table’s structure and applications.^7^ While mnemonic devices have been used to support memorization, they often fail to provide a holistic understanding of the periodic table’s significance.^8^

To address these challenges, various innovative approaches have been explored, as reviewed by Borges^9^ and Stojanovska.^10^ These include collaborative group learning,^11,12^ crossword puzzles,^13−15^ digital periodic tables,^16,17^ descriptive approaches,^18^ games,^19−28^ toy bricks,^29−32^ hands on activities,^33,34^ and even principal component analysis.^35−37^ Among these, the concept of serious games and gamification have emerged as promising tools, offering an engaging and interactive alternative to traditional teaching methods.^13,19,38−42^

This work introduces *Snakeleev*, an educational video game inspired by the iconic Snake game, reimagined as a tool for exploring the periodic table of chemical elements. In *Snakeleev*, players guide a growing snake to *“consume”* chemical elements, identified by their symbols, and classify them into thematic *“diets”* that reflect both traditional category (e.g., metals, nonmetals, and transition metals) and real-world applications such as critical raw materials,^43,44^ smartphone,^45−47^ fusion power plant,^48^ radioactivity,^49^ and potentially toxic trace elements (PTEs).^50,51^ These diets, along with unconventional ensembles (e.g., elements linked to scientists,^52^ biology,^53^ geology,^54,55^ or even astronomy — such as elements produced through white dwarf explosion, rather than in massive star explosion), highlight the periodic table’s relevance to contemporary challenges and interdisciplinary contexts.

*Snakeleev* challenges players to recognize chemical elements by their atomic symbols and classify them into meaningful, real-world categories, reinforcing their understanding of the periodic table of elements. The gameplay enhances pattern recognition and decision-making, akin to the benefits observed in flashcards,^56,57^ while bridging the gap between the atomic world and practical applications. Unlike rote memorization exercises, the game fosters retention in an engaging, low-pressure environment and encourages exploration beyond standard periodic table groups. Educators can integrate *Snakeleev* into dynamic and engaging lessons as a versatile tool for interactive learning. At the end of each game, players receive insights related to their chosen diet, deepening their appreciation of the periodic table’s relevance in science and society.

To assess the game’s effectiveness and reception, *Snakeleev* was tested with high school students (second-year classes) using the “*Elements of a smartphone*” diet. A survey conducted after the gameplay revealed that the game was well-received by students, who found it both enjoyable and educational. The results highlight S*nakeleev*’s potential as an innovative teaching tool that combines learning with entertainment.

Developed primarily in Python^58^ (as in the first version,^59^ themed around Norse mythology, particularly the depiction of Thor,^60,61^ see Appendix A) and later adapted into a browser-based game, *Snakeleev* requires no installation or programming expertise, making it highly accessible. This flexibility allows the game to be used in classrooms or on personal devices, including smartphones, significantly broadening its reach and utility even during leisure times. By making *Snakeleev* free and open source, with its code available as both a Python package^62,63^ and an HTML-JavaScript implementation^64,65^ on GitHub, the project empowers educators and students to customize and expand the game, fostering active learning and engagement with both chemistry and programming.

Finally, *Snakeleev* is rooted in the broader educational framework of the project *“Change the Game: Playing to Prepare for the Challenges of a Sustainable Society”*,^66^ which aims to raise awareness of sustainability issues through the design and testing of serious games.^67,68^ In this context, *Snakeleev* not only promotes chemistry education but also encourages critical thinking about the role of science in addressing real-world challenges.

## Game Design

**Figure 1:**
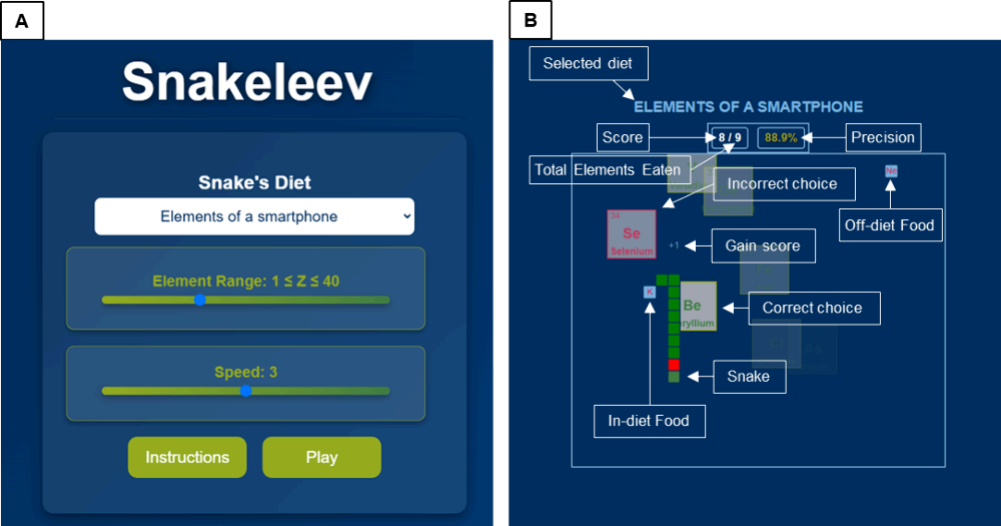
Screenshots of *Snakeleev*’s user interface and gameplay mechanics. (A) The setting page, where players can: choose from 41 thematic “*diets*” of elements, such as “*Elements of a smartphone*”; set the maximum atomic number (*Z*) of elements appearing in the game (ranging from 1 to 118); and select the snake’s speed, ranging from 1 (slowest) to 5 (fastest). Players can also access gameplay instructions by clicking the “Instructions” button. (B) The gameplay interface highlights key components: the selected diet (top) and the score (center). Correctly consumed elements are displayed in green, increasing the score, while incorrectly consumed elements appear in red and decrease the score. The snake grows with each consumed element, and its “*history*” visually records correct (green) and incorrect (red) choices. Players can swap the two elements’ position by pressing the spacebar. while food (elements) appears randomly on the game field.

The design of *Snakeleev* evolved to enhance playability and ensure a clear distinction between elements belonging to the selected diet and those that do not. Originating from an earlier version^59,62^ inspired by Norse mythology (see Appendix A), the game introduced several key features to improve the experience and minimize potential frustration during gameplay:

1. Speed selection: on the first game page (Figure 1a), players can select the snake’s speed from five levels, ranging from 1 (slowest) to 5 (fastest). This customization allows players to tailor the game to their comfort, experience, and skill level, fostering a more inclusive and adaptable learning environment.
2. Element quantity: below the speed slider (Figure 1a), players can set the maximum number of elements that will appear during gameplay. This feature ensures the challenge remains balanced and manageable for players with varying levels of familiarity with the periodic table, while also offering the option to explore transuranic and superheavy elements for advanced users seeking a deeper dive into the periodic table.^69^
3. Wrap-around effect: to maintain continuous gameplay and avoid unnecessary frustration, the *wrap-around effect* was introduced. Instead of ending the game when the snake collides with the screen’s borders, the snake passes through one side of the screen and reappears on the opposite side. This effect supports better focus and fluid gameplay.
4. Collision Detection: game mechanics were fine-tuned to enhance user experience by only registering collisions that occur perpendicularly. If the snake moves parallel to itself and the player attempts to direct it into its own body, the command is ignored. This feature minimizes disruptions caused by minor directional missteps, allowing players to maintain focus on learning.
5. Optimized element placement: chemical elements do not spawn near the borders, ensuring they remain easily visible and legible.
6. Highlighted element information: after consuming an element, its name and symbol are briefly displayed in an enlarged format (Figure 1b). This feature facilitates reading and retention of the element without distracting the player from controlling the snake. The impact of this feature varies depending on the selected speed level: at speed level 5, players must be highly confident with the keyboard, game mechanics, and rapid reading to keep up with the fast-paced gameplay. In contrast, at speed level 1, players can take their time to read the displayed information without the pressure of managing the snake’s movement, making the game accessible to all skill levels.
7. Color emphasis for eaten elements and snake growing (Figure 1b): Consumed elements are highlighted using green or red colors to indicate whether they belong to the selected diet (green) or not (red), reinforcing the distinction visually. Additionally, the corresponding segment of the snake grows in green or red depending on whether the element belongs to the diet. This visual feedback system creates a dynamic and intuitive “*history*” of correct and incorrect choices, allowing players to visually track their performance and decisions through the snake’s color-coded growth pattern.
8. Directional movement based on element classification: the visual feedback extends to the upward or downward motion of the elements after consumption (Figure 1b). If an element belongs to the selected diet, its card (containing its atomic number, symbol, and name) moves upward; conversely, it moves downward if it does not belong to the selected diet. This motion reinforces the classification system and provides additional cognitive feedback during gameplay.
9. Pause function: players can pause the game at any time by pressing “P”.
10. An initial attempt to play *Snakeleev* on a smartphone was made by enabling players to control the snake using the touch screen.

These design enhancements collectively create a smoother and more engaging gaming experience, allowing players to focus on memorizing elements and understanding their classification without being hampered by technical frustrations or gameplay mechanics. As detailed in the Supporting Information, these improvements were implemented at different stages, ultimately culminating in the current structure of *Snakeleev* as described in the following subsections. In this version, repeated exposure, active recall, visual feedback, and pattern recognition strengthen familiarity with element names and symbols, enhancing both memorization and classification over time. A detailed analysis of learning effectiveness, based on pre- and postgameplay test results, is provided in the Testing section.

### Rules and Gameplay

Before starting the game, players can access the instructions by clicking on the dedicated button (Figure 1a). To control the snake in *Snakeleev*, players can use either the arrow keys or the A, D, S, W keys, with the latter being recommended for left-handed players. These directional controls enable players to navigate the snake throughout the game environment, guiding it to consume the elements that they believe belong to the selected diet. Unlike the classic *Snake* game, *Snakeleev* displays two elements at a time above the game area (see Figure 1b). One of these elements belongs to the selected diet, while the other does not. As the snake consumes an element, it grows—regardless of whether the consumed element is correct or incorrect—increasing the challenge of avoid self-collision. This mechanic closely mirrors the mechanics of the original Snake game. However, when the snake consumes a correct element, a green segment is added to its body, and the score increases by one. Conversely, consuming an incorrect element adds a red segment, but the remains unchanged. The player’s score is prominently displayed at the center above the game area and below the name of the selected diet (Figure 1b). The score is formatted as “score/total elements consumed”, providing a real-time indication of the player’s progress. Additionally, the precision percentage—calculated as shown in eq 1—is displayed next to the score, offering an intuitive measure of accuracy
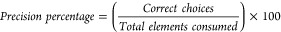
1where correct choices correspond to the player’s score. This percentage is visually represented using a gradient color scale, transitioning from red (low accuracy) to green (high accuracy), providing immediate feedback on performance. The combination of numerical and color-coded feedback provides an intuitive and engaging way to track learning progress in elements classification while maintaining the game’s fast-paced and interactive nature. Players can press the space bar to swap the positions of the two elements. This mechanic allows players to (i) speed up gameplay by positioning the correct element closer and (ii) consume the correct element when changing direction is not feasible. No mechanism was introduced to prevent the consumption of incorrect elements that appear directly in front of the snake. In such cases, the player may not have enough time to recognize that the snake is about to consume the wrong element and press the space bar in time. This apparent bug was intentionally left in the game to introduce an element of randomness that can impact the final score. This kind of unpredictable event adds an element of chance, making the gameplay more dynamic and slightly less deterministic. Hypothetically, in the later stages of the game, when the snake has filled a significant portion of the playing area, such events will occur more frequently. Initially, success in these situations will depend on the player’s reflexes; however, as the snake grows longer, chance will become the predominant factor. Ultimately, this randomness will play a key role in allowing players to surpass previous high scores.

### Elemental Diets: Scientific Themes and Interdisciplinary Connections

*Snakeleev* offers an extensive selection of 41 implemented diets, providing an educational and engaging experience that spans various scientific disciplines and beyond (see Table 1). Each diet has been carefully curated to reflect essential aspects of element, classifications, their occurrence in substances,^70^ and real-world applications. Many of these themes are inspired by the author’s research activities, such as the development of plasma-facing materials for fusion power plants (e.g., “*Elements considered safety (grades A-E) in the first wall of fusion power plants*” and “*Radioactive elements (U–Th decay series)*”),^71^ piezoelectric membranes for wastewater microfiltration (“*Potentially toxic trace elements (PTEs)*”),^72^ spinel ferrites (“*Ferromagnetic elements*”)^73,74^ and ultrahigh temperature ceramic matrix composites (“*Ultra-high temperature metals*”).^75−77^ For instance, the “*Elements of life*” diet highlights the fundamental building blocks of biological systems, as famously discussed by Isaac Asimov,^54^ underscoring the significance of elements in biology and the chemistry of life. Similarly, the “*Elements of DNA*” diet delves into the genetic fundamentals of life.^53^ These diets not only promote understanding of materials science but also have significant implications in other scientific disciplines such as biology and medicine.

**Table 1:** Implemented Diets with Correlated References

Diet name	Elements included	ref.
Critical elements	Sb, Ba, Al, Be, Bi, B, Co, F, Ga, Ge, Hf, In, Li, Mg, Nb, P, Sc, Si, Sr, Ta, Ti, W, V	(43), (44)
Elements of a smartphone	H, Li, Be, B, C, O, F, Na, Mg, Al, Si, P, S, Cl, K, Sc, Ti, V, Cr, Mn, Fe, Co, Ni, Cu, Zn, Ga, Ge, As, Br, Rb, Sr, Y, Zr, Nb, Mo, Ru, Rh, Pd, Ag, Cd, In, Sn, Sb, Te, Ba, La, Ce, Pr, Pm, Sm, Eu, Gd, Tb, Dy, Ho, Er, Tm, Yb, Lu, Hf, Ta, W, Os, Ir, Pt, Au, Hg, Pb, Bi	(45−47)
Elements of life	O, C, H, N, P, Ca, S, K, Na, Cl, Mg, Fe, Zn, Cr, Co, Cu, Mn, Mo, Ni, V, Si, B, Se, F, I, Br	(54)
Elements of DNA	C, H, O, N, P	(53)
Elements essential for man	H, C, N, O, F, Na, Mg, Si, P, S, Cl, K, Ca, V, Mn, Fe, Co, Ni, Cu, Zn, Se, Mo, Sn, I	(80)
Elements used in therapy	H, He, Li, B, C, N, O, F, Na, Mg, Al, Si, P, S, Cl, Ar, K, Ca, Ti, V, Cr, Mn, Fe, Co, Cu, Zn, Ga, As, Se, Sr, Mo, Ru, Pd, Ag, Sb, Xe, La, Ce, Ta, Os, Pt, Au, Bi	(80)
Elements used in diagnosis	He, F, Cu, Ga, Ge, Rb, Y, Zr, Tc, In, I, Xe, Ba, Gd, Tl	(80)
Medical radioisotopes	Sr, Y, Rh, Pd, I, Cs, Sm, Ho, Lu, Re, Ir, Bi, At, Ra, Ac	(80)
Radioactive elements (U–Th decay series)	U, Th, Pa, Ra, Rn, Po, Pb, Bi, Pu, Ac, Tl, Am, Np	(49)
Elements considered safety (grades A-E) in the first wall of fusion power plant	H, He, Li, Be, B, C, N, O, F, Ne, Mg, Al, Si, P, Cl, Ar, K, Ca, Sc, Ti, V, Cr, Fe, Co, Ni, Cu, Ge, Se, Kr, Sr, Y, Ru, Sn, Te, I, Xe, Cs, Ba, Ce, Nd, Sm, Dy, Yb, Lu, Tl	(48)
Potentially toxic trace elements (PTEs)	Pb, Hg, Cr, Cd, As, Co, Cu, Ni, Zn, Ag, Sb, Fe, Mn, Zr, Se, Sr, Al, F, U, La, Pr, Au	(50), (51)
Toxic trace elements in dried mushrooms	As, Cd, Hg, Pb	(81)
Elements dedicated to scientists	Ga, Ge, Sm, Gd, Cm, Es, Fm, No, Lr, Rf, Sg, Bh, Mt, Rg, Og	(52)
Elements with names of Latin derivation	B, C, F, Na, Al, Si, S, K, Ca, Sc, Mn, Fe, Cu, Ga, Ge, Rb, Ru, Pd, In, Sn, Sb, Te, Cs, La, Ce, Pm, Eu, Ho, Tm, Lu, Hf, Ta, Ir, Au, Hg, Pb, Bi, Po, Rn, Ra, Np, Cm, Hs	(54), (55)
Elements with names of Greek derivation	H, He, Li, Be, N, O, Ne, P, Cl, Ar, Ti, Cr, Co, As, Se, Br, Kr, Nb, Mo, Tc, Rh, Ag, Cd, Sb, I, Xe, Ba, La, Pr, Nd, Dy, Os, Tl, Bi, At, Ac, Pa, U, Pu	(54), (55)
Elements named after geographical locations and celestial bodies	Mg, Sc, Mn, Ga, Ge, Se, Sr, Y, Nb, Tc, Ru, Pd, Cd, Te, Eu, Tb, Ho, Er, Tm, Yb, Lu, Hf, Re, Bi, Po, Fr, U, Np, Am, Bk, Cf, Db, Hs, Ds	(54), (55)
Elements with names not derived from Latin or Greek, nor from cities or countries	V, Co, Ni, Zn, Zr, Sb, W, Pt, Bi, Th	(54), (55)
Elements known since antiquity	C, S, Fe, Cu, As, Ag, Sn, Sb, Au, Hg, Pb	(78), (79)
Elementary substances in solid state at standard temperature and pressure	Li, Be, B, C, Na, Mg, Al, Si, P, S, K, Ca, Sc, Ti, V, Cr, Mn, Fe, Co, Ni, Cu, Zn, Ga, Ge, As, Se, Rb, Sr, Y, Zr, Nb, Mo, Tc, Ru, Rh, Pd, Ag, Cd, In, Sn, Sb, Te, I, Cs, Ba, La, Ce, Pr, Nd, Pm, Sm, Eu, Gd, Tb, Dy, Ho, Er, Tm, Yb, Lu, Hf, Ta, W, Re, Os, Ir, Pt, Au, Tl, Pb, Bi, Po, At, Fr, Ra, Pa, U, Np, Pu, Am, Cm, Bk, Cf, Es, Fm, Md, No, Lr	(78), (79)
Elementary substances in liquid state at standard temperature and pressure	Hg, Br	(78), (79)
Elementary substances in gas state at standard temperature and pressure	H′′, He, N, O, F, Ne, Cl, Ar, Kr, Xe, Rn	(78), (79)
Ferromagnetic elements	Fe, Co, Ni, Gd, Tb, Dy, Ho, Er, Tm	(82), (83)
Ultrahigh temperature metals	W, Re, Ta, Os	(78), (79), (84)
Classic diets: Metals, Nonmetals, Elements of group I (Hydrogen and alkali metals), Elements of group II (Alkaline earth metals), Elements of group XV (Pnictogens), Elements of group XVI (Chalcogens), Elements of group XVII (Halogens), ′′Elements of group XVIII (Noble gases), Lanthanides, Actinides, Transition metals, Post-transition metals, ′′Metalloids, Reactive nonmetals, s-block elements, p-block elements, d-block elements, f-block elements		(78), (79)

Many diets integrate connections with history, geography, and linguistics, further enriching the gaming experience. For example, the “*Elements dedicated to scientists*” diet celebrates historical figures who contributed to the discovery and understanding of chemical elements, integrating elements of the history of science into the game’s storyline. Likewise, the *Elements with names of Latin or Greek derivation* diet explores the linguistic and cultural origins of element nomenclature, encouraging players to discover the interplay between science, history, and culture. These themes reflect the author’s classical education, which included the study of Latin and Greek in high school.

Some diets delve into themes with significant political and economic implications. The “*Critical elements*” diet^43,44^ explores the global dependency on rare elements and their role in key industries such as technology (e.g., smartphones^47^) and sustainable energy production, including fusion power plant.^48^ These diets raise awareness about supply chain security, resource sustainability, and equity in resource access. Diets with strong environmental education potential, such as “*Potentially toxic trace elements (PTEs)*” and “*Elements of a smartphone*”, can be used to sensitize students about the importance of proper waste disposal, particularly electronic waste (RAEE). These themes also highlight the fascinating concept that “*you carry a periodic table in your pocket*” through everyday devices.

*Snakeleev* also incorporates groups tied to the periodicity of the periodic table.^78,79^ The game’s modular design allows for seamless integration of new diets, offering opportunities to connect chemical elements with topics in natural sciences, history, geography, narrative, and political and economic issues. This flexibility enhances the game’s educational value and supports comprehensive learning about the elements and their applications. Further diets can be designed to align with specific classroom topics or scientific themes. For example,

- Astronomy: diets focusing on elements produced through white dwarf explosions, massive star explosions, or merging neutron stars.
- Applications: diets focused on materials used in batteries, screens, or energy storage technologies.
- Specific Materials: diets based on alloys of Fe, Al, Mg, and other industrially relevant materials.
- History and technology: diets where it is possible to select the year of discovering.
- Other chemical features of the periodic table such as electronegativity (Pauling scale), oxidation state.

By fostering curiosity and encouraging exploration, *Snakeleev* provides a dynamic platform to connect chemical knowledge to diverse scientific, historical, and cultural contexts.

### Game Over: Keep to Stay Hungry, Stay Periodic!

**Figure 2:**
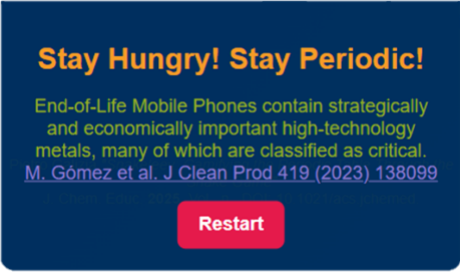
Screenshot of the game over window *(*“*stay hungry, stay periodic*” window). In this example a message correlated to the “*Element of a smartphone*” diet is printed.

While playing *Snakeleev* enhances players’ ability to classify and recognize elements, the game over screen presents an opportunity to impart a meaningful message or insightful curiosity about the selected diet’s theme. Instead of the conventional “GAME OVER” text, the screen displays the uplifting phrase “*Stay Hungry! Stay Periodic!*”, a creative adaptation of Steve Jobs’ iconic and inspiring quote, “*Stay hungry, stay foolish*”. This serves as a motivational motto, encouraging players to maintain curiosity and commitment to periodic exploration even in the face of complexity. To make this moment more impactful, the screen also showcases a random phrase or fact related to the selected diet see Figure 2). These messages are carefully curated and can include interesting trivia about the elements within the chosen diet or excerpts from literary works that reference the elements in the selected diet, such as *The Periodic Table* by Primo Levi or *Uncle Tungsten* by Oliver Sacks. This thoughtful design transforms the Game Over moment into an educational and reflective experience.

## Testing

This activity was conducted with two second-year classes from a scientific high school (similar to a college-preparatory high school with a focus on STEM subjects), with 27 and 24 students, respectively. The study aimed to assess *Snakeleev*’s effectiveness in helping students learn element symbols, and name, and classification within a thematic context. The “*Elements of a Smartphone*” diet was selected to make the periodic table more relatable by linking it to an object familiar to all students, thereby increasing engagement and relevance. The activity involved two tests: (A-test) students were tasked with writing down the full names of the first 40 elements of the periodic table (from hydrogen to zirconium), while (C-test) required them to identify which of these elements they believed were present in smartphones. Students circled the elements they thought were present in smartphones and crossed out those they believed were not. This test was administered both before and after playing *Snakeleev* for 10 min to assess their prior knowledge and learning outcomes. After repeating this procedure twice, students were asked to complete a survey to share their opinions on the game and evaluate their overall satisfaction. Further details and preliminary activities are provided in Supporting Information.

### Student Feedback and Satisfaction Survey

**Figure 3:**
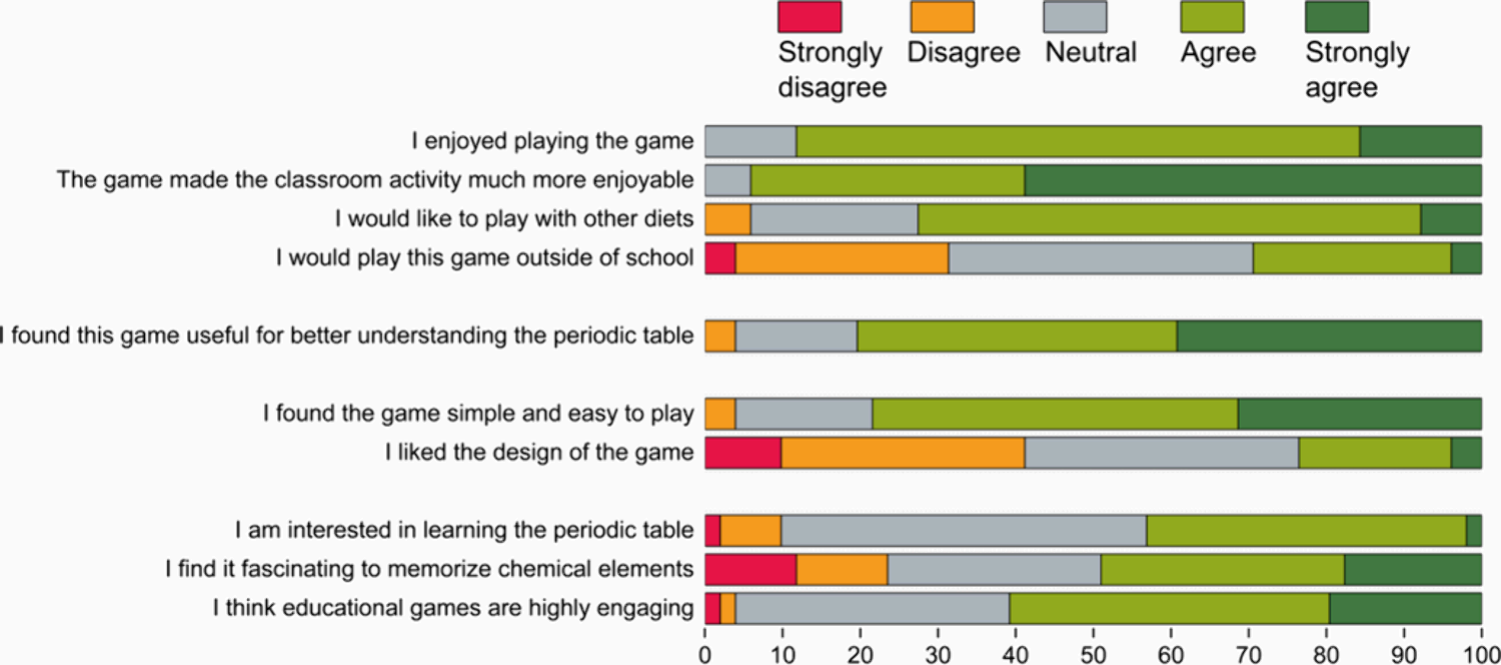
Bar chart displaying the percentage distribution of responses on a 5-point Likert scale, with red indicating “strongly disagree,” orange “disagree,” gray “neutral,” light green “agree,” and dark green “strongly agree.” The survey involved a sample of 51 students participating in the classroom activity.

The survey summarized in Figure 3 presents students’ perceptions and feedback on the educational game *Snakeleev*, supplemented by an additional survey with five Yes/No questions and three open-ended questions (see Supporting Information). The results highlight a strong positive response to the game, emphasizing both its educational value and its potential for wider use. Over 90% of students responded affirmatively to having learned new elements and symbols, found the game helpful in identifying the elements included in a selected diet, and stated they would recommend the game to a friend or classmate. Additionally, 82% expressed interest in playing the game on their smartphones, demonstrating its accessibility and adaptability as a learning tool.

According to Figure 3, an overwhelming majority of students found *Snakeleev* enjoyable and engaging. Specifically, 73% agreed, and 16% strongly agreed, with the statement, “I enjoyed playing the game,” while 35% agreed and 59% strongly agreed that “the game made the classroom activity much more enjoyable.” These results underscore the game’s ability to create a dynamic and stimulating learning environment. Furthermore, more than 70% of respondents expressed interest in exploring the game further with additional “diets”, highlighting its replicability and potential for future expansion. Interestingly, a more neutral distribution was observed when students were asked whether they “would play this game outside of school.” Some students clarified that they viewed the game primarily as a didactic tool, useful specifically for studying the periodic table. Nonetheless, the overall level of enthusiasm, as claimed by educators and teachers, suggests that the game’s appeal could extend beyond the classroom, making it a viable option for informal, independent learning. Comments such as “You learn, and it’s surprising,” “Even without trying, you automatically learn the periodic table quickly,” and “It definitely saves me hours of studying” highlight how *Snakeleev* effectively integrates education with entertainment, enabling students to learn in an enjoyable and relaxed manner. The game’s intuitive and user-friendly design also received praise. Most students found the game “simple and easy to play,” with 47% agreeing and 31% strongly agreeing. Open-ended responses further emphasized the game’s simplicity, creativity, and its ability to combine mental challenges with educational content. Students particularly appreciated how the game uses visual and interactive elements to make learning engaging and accessible. However, the survey also revealed areas for improvement. Some students expressed frustration with the absence of a pause button, and limited visibility of certain elements on the screen. They suggested enhancements such as improving the graphics, introducing customizable features (e.g., snake colors), and adding more interactive elements like leaderboards, multiplayer options, and personal records. These suggestions align with responses to the question about the “design of the game,” where a distribution skewed by 17% toward disagreement reflects some dissatisfaction with the visual and functional aspects of the game.

The survey also revealed broader insights into students’ attitudes toward educational games. While many students agreed that they find educational games highly engaging and expressed interest in learning the periodic table, not all were fascinated by the process of memorizing chemical elements, symbols, and atomic numbers. Nevertheless, the fact that 41% agreed and 39% strongly agreed that they “found this game useful for better understanding the periodic table”—after only 20 min of gameplay—underscores the value of gamified learning approaches. These results corroborate *Snakeleev*’s potential to make learning elements and their classification more accessible and enjoyable. Overall, the feedback illustrates that *Snakeleev* successfully captures students’ interest and enhances their understanding of chemistry in an innovative way. By addressing the suggested improvements (see the “*Future Work and Potentialities for Elements’ Snake and Beyond*” section and the “*Development of Snakeleev: From Python Package to Browser Game*” section in the Supporting Information), the game could become an even more effective educational resource, further bridging the gap between enjoyable gameplay and meaningful learning.

### Effectiveness Assessment

**Figure 4:**
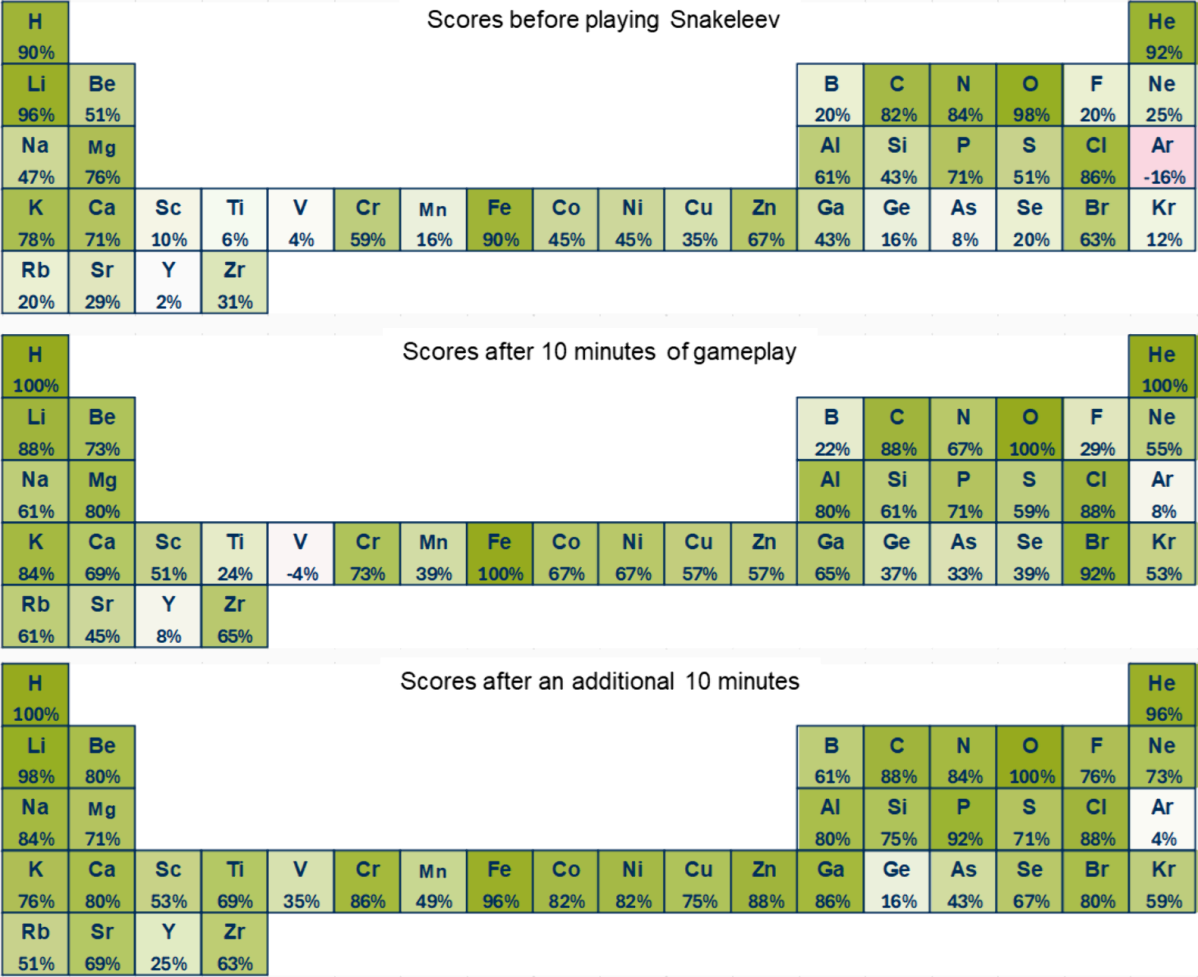
Heat maps illustrating the performance of 51 students in the A-test (associating element symbols with their names) before playing *Snakeleev*, after 10 min of gameplay, and after an additional 10 min. The green shading represents positive scores (from 0 to +1), while red shading indicates negative scores (from 0 to −1). Scores were calculated individually by assigning +1 for each correct response and – 1 for each incorrect response. The total for each element was divided by the number of students (51) and converted into percentages.

The largely positive feedback from students regarding *Snakeleev* was substantiated by quantitative data on its learning effectiveness, as measured through the A- and C-tests. The heat maps in Figure 4 illustrate the average scores for each element across all 51 students at three time points: before gameplay, after 10 min, and after an additional 10 min. Scores were calculated by assigning +1 for each correct response and – 1 for each incorrect response, dividing the total by 51, and converting it into percentages.

Before gameplay, significant variability in scores was observed, with only 18 elements achieving an average score above 0.5 and just 10 elements exceeding 0.75. After 10 min of gameplay, notable improvements were evident for most elements, including those with initially low or negative scores. For instance, vanadium’s score rose from −4% to 24%, and argon improved from −16% to 8%. The number of elements with average scores above 0.5 and 0.75 increased to 29 and 11, respectively.

After an additional 10 min of gameplay, these gains were further reinforced. Elements like vanadium improved to 35%, while gallium saw a dramatic increase from 43% before gameplay to 86%. Familiar elements, such as hydrogen and helium, consistently maintained high or perfect scores throughout the testing process. By this stage, 34 elements achieved an average score above 0.5, and 23 exceeded 0.75.

**Figure 5:**
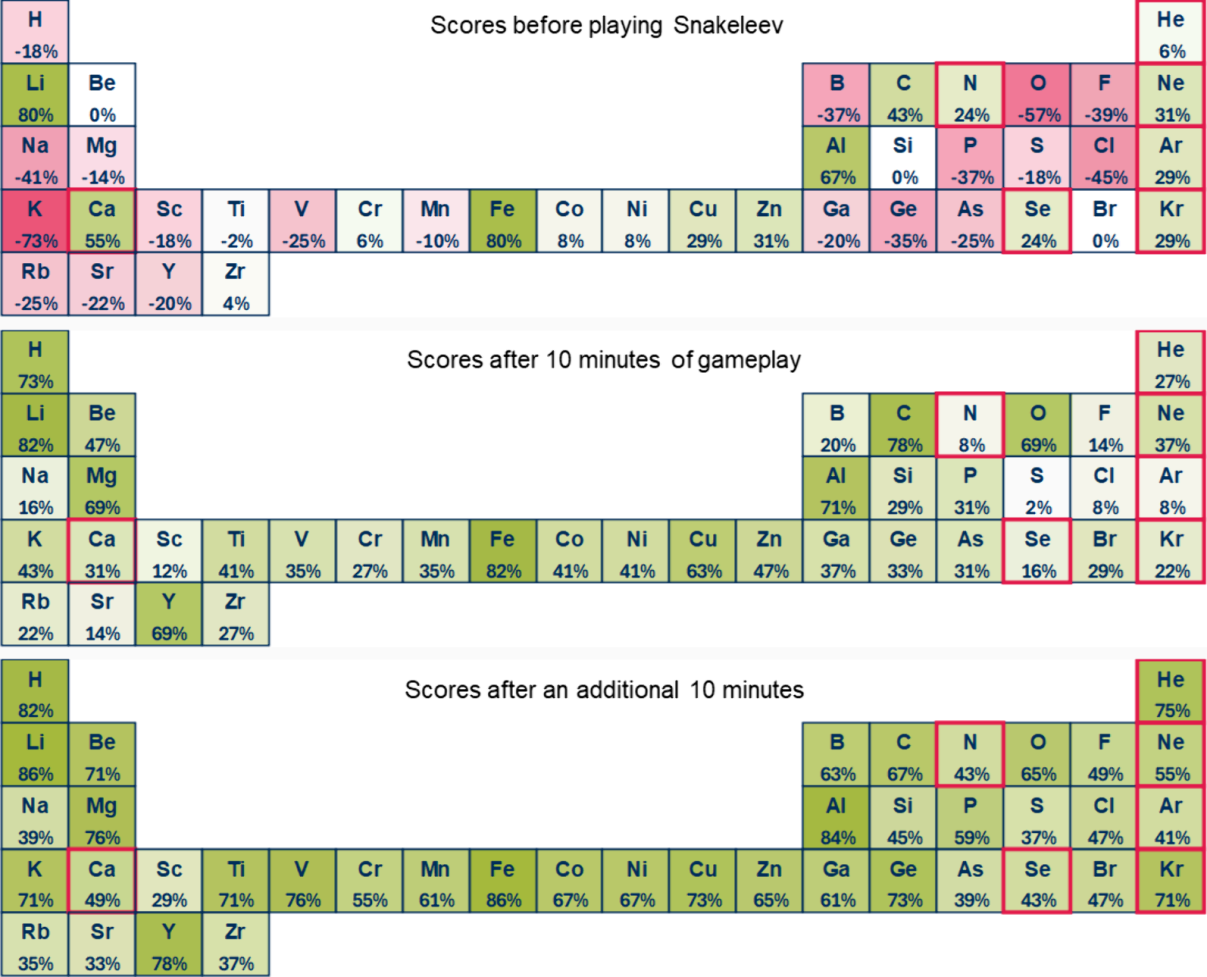
Heat maps illustrating the performance of 51 students in the C-test (classifying elements as part of the selected diet: “Elements of a Smartphone”) before playing *Snakeleev*, after 10 min of gameplay, and after an additional 10 min. The green shading represents positive scores (from 0 to +1), while red shading indicates negative scores (from 0 to −1). Scores were calculated individually by assigning +1 for each correct response and – 1 for each incorrect response. The total for each element was divided by the number of students (51) and converted into percentages. Red-outlined elements represent those not included in the selected diet.

The effectiveness of *Snakeleev* was particularly evident in the results of the C-test, which assessed students’ ability to classify elements as present or absent in a smartphone (Figure 5). Before gameplay, the results showed a widespread lack of awareness, with only 10 elements achieving an average score above 0.25. After 10 min of gameplay, this number rose significantly to 28 elements and, after an additional 10 min, every element in the test was correctly classified by the majority of students.

Gameplay helped students recognize the broader diversity of elements involved in smartphones, revealing misconceptions and highlighting learning opportunities. For instance, lithium—critical for batteries—and cobalt—central to coltan mining and its associated social and environmental issues—were initially overlooked but were more accurately identified after gameplay. Similarly, students initially excluded elements like carbon and hydrogen, which are essential to plastic components, and oxygen, which plays a critical role in materials such as ceramics used in capacitors and insulators. Silicon, a cornerstone of electronics and glass manufacturing, was also surprisingly omitted in many cases during the pregame phase. These gaps revealed a lack of foundational knowledge about materials science, which gameplay helped to address by encouraging students to make connections between the elements and their practical applications. Beyond its scientific insights, the exercise spurred broader discussions about the economic, environmental, and geopolitical implications of material use. Elements like lithium and cobalt, while essential for smartphone functionality, also raise ethical and sustainability concerns due to the environmental degradation and labor exploitation associated with their extraction, particularly in regions involved in coltan mining. These reflections underscored the interconnectedness of materials science with pressing global issues, fostering critical thinking about the lifecycle of technological devices, the importance of recycling, and the broader consequences of material consumption.

**Figure 6:**
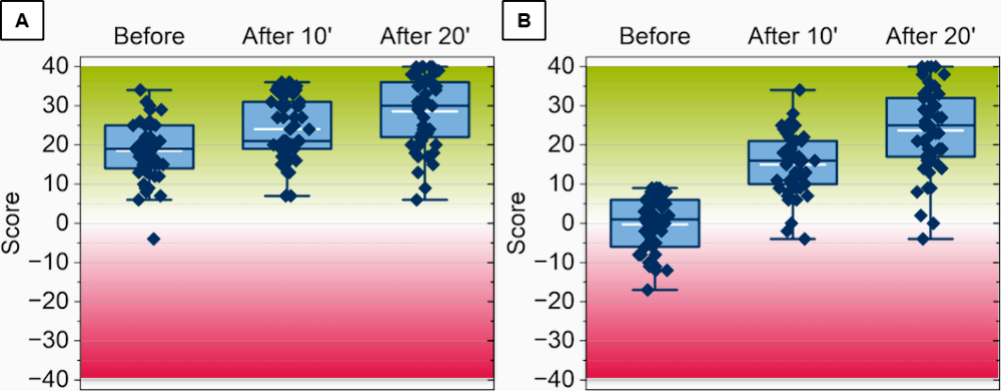
Performance of 51 students in the A-test and C-test before playing *Snakeleev*, after 10 min of gameplay, and after an additional 10 min. (A) The A-test evaluated students’ ability to associate element symbols with their corresponding names, while (B) the C-test assessed their ability to identify elements linked to the “Elements of a Smartphone” diet. Scores were calculated individually by assigning +1 for each correct response and – 1 for each incorrect response. Green shading covers the scores from 0 to 40, red shading covers negative scores from 0 to −40. The boxes represent range from 25 percentile to 75 percentile, with the horizontal blue line dividing the scores into two equal groups. The white line within each box indicates the mean percentage of the scores.

The significant effect of *Snakeleev* on students’ performance is clearly illustrated in the boxplot (Figure 6). The mean scores increased from 18.5 ± 1.0 to 24.0 ± 1.1 and 28.5 ± 1.2 after 10 and 20 min for the A-test, and from – 0.3 ± 1.0 to 15.0 ± 1.2 and 23.7 ± 1.5 for the C-test. Remarkably, after 20 min of gameplay, 10% (A-test) and 8% (C-test) of students achieved the maximum score of 40, compared to just 2% and 8% before gameplay, when the highest scores were 34 (A-test) and 9 (C-test). To assess the statistical significance of score changes across the three conditions (Before, After 10’, After 20’), the Friedman test was performed. Due to the non-normal distribution of scores (as confirmed by the Shapiro-Wilk test, see Table S5 of Supporting Information), this nonparametric approach was chosen.^85,86^ The Friedman test results (χ^2^ = 214 for A-tests; χ^2^ = 163 for C-tests, *p* < 0.0001) strongly indicate significant differences among the conditions.^87,88^ Posthoc analysis using Dunn’s Test and Wilcoxon-Nemenyi-McDonald-Thompson Test confirmed significant improvements in scores after both 10 and 20 min of gameplay.

To quantify the effect size, Cohen’s d was calculated for each pairwise comparison. According to Cohen,^89^ effect sizes are classified as small (d ≈ 0.2), medium (d ≈ 0.5), and large (d ≈ 0.8). Additionally, values above 1.2 are considered very large, while those exceeding 2.0 are classified as huge.^90^ The effect was very large for A-tests (d = 1.23) and huge for C-test (d = 2.67) when comparing scores after 20 min of gameplay with baseline (Before). These values suggest a strong impact of *Snakeleev* on improving scores, particularly for the C-test, which measures classification skills. While the improvement was more modest between 10 and 20 min (d = 0.54 for A-test; d = 0.94 for C-test), it was still substantial. The largest gains occurred within the first 10 min (d = 0.73 for A-test; d = 2.1 for C-test). This pattern suggests an “instantaneous” learning effect, with rapid initial improvements followed by smaller, continued gains. The C-test benefited more from gameplay compared to the A-test, likely because students were less familiar with the elements related to smartphones. Therefore, the observed differences reflect baseline preparedness rather than a differential effectiveness of the game. Combined with students’ positive survey responses and visible enjoyment during gameplay, these results highlight the potential of *Snakeleev* as an engaging and educational tool for learning about elements and related scientific topics (e.g., materials science, circular economy, or chemistry history). Future research should further validate these findings by comparing the game’s effectiveness with alternative study approaches.

Summary of Effectiveness Assessment’s Key Findings in Simple Terms

- **Quick learning**: students improved their ability to recognize and classify elements after just 10 min of gameplay.
- **Higher scores:** average scores increased from 18.5 to 28.5 in the A-test (matching symbols to names) and from −0.3 to 23.7 in the C-test (classifying elements in a smartphone).
- **Rapid early progress:** most learning occurred in the first 10 min, with steady but smaller gains afterward.
- **Improved element recognition:** in the A-test, the number of elements with an average score above 75% nearly doubled (from 18 to 34). In the C-test, most students correctly classified all elements after 20 min of gameplay.
- **Real-world awareness:** students linked elements like lithium, cobalt, and to smartphones, fostering awareness of sustainable technology.
- **Statistically significant improvements:** score gains were confirmed through statistical analysis, indicating meaningful learning outcomes.

## Future Work and Potentialities for Elements’ Snake and Beyond

Building on the success of the game’s development and the promising results from testing, further improvements are being explored. These enhancements aim to refine the technical foundation, enrich gameplay, and expand the game’s reach and educational applications.

### Player Records and Leaderboards

A frequently requested improvement is the ability to save player records, along with their nickname. Personal score tracking, combined with a top-ten leaderboard for each diet in a secondary could foster a sense of competition. The leaderboard would be displayed in a secondary window, motivating players to achieve higher scores and secure a spot in the rankings.

### Statistical Feedback, Learning Analytics, and Context-Specific Feedback

Providing players with statistical feedback could greatly enhance the learning experience by helping them track their accuracy and progress. Indicators such as precision in selecting correct elements, frequency of mistakes, and recall improvements would offer valuable insights into their learning trajectory. A heat map of the periodic table, highlighting frequently missed elements (similar to Figures 4 and 5), would serve as a visually engaging representation of player performance.

To further personalize the learning experience, context-specific feedback will be introduced. At the end of each game, players will receive detailed messages about their mistakes, explaining why certain elements do or do not belong to the selected diet. This feature will transform errors into learning opportunities, reinforcing key concepts. By integrating these statistical and feedback mechanisms, Snakeleev will encourage active learning, allowing players to self-assess and gradually improve their understanding of the periodic table.

### Customizable Diets and User-Generated Content

Recognizing the complexity and occasional ambiguity in classification—particularly in interdisciplinary contexts such as chemistry, history, and language—the game will introduce a “*Create Your Own Diet*” feature. This will allow players and educators to define their own thematic groupings of elements, fostering active participation, reasoning, and exploration. Educators could use this as a classroom activity or even as a homework assignment to encourage deeper engagement with classification challenges. This approach aligns well with constructivist educational theories by prompting players to critically analyze and justify their categorizations. If feasible, players or educators could share and exchange custom diets, making learning more collaborative and discussion-driven. By enabling users to construct, justify, and potentially share their custom diets, *Snakeleev* will further encourage exploration, engagement, and deeper interaction with the periodic table beyond rigid, predefined categories. Future iterations may also introduce a community-driven repository where educators and students can browse, rate, and discuss various custom diets, fostering a more interactive and social learning experience.”

### New Game Mode

- **Level-based mode:** players start at Level 0 with a small subset of elements (e.g., the first 8) and progress to higher levels by performing a set number of consecutive correct actions (eating or skipping elements). In each subsequent level, additional elements are introduced (e.g., 10 new elements per level), with the ultimate challenge being Level 11, where all 118 elements are in play.
- **Test mode:** players take a test before and after playing the game. This would allow for the same calculations presented in the “Testing” section to be performed, facilitating easier monitoring of the game’s effectiveness.
- **Weighted scoring system:** a scoring system could incorporate parameters like element abundance in specific context (e.g., smartphone components or Earth’s crust or atmosphere) or physical properties like decay rates. This could pave the way for a comprehensive figure of merit that integrating multiple parameters.
- **Game configuration options:** players could gain the option to enable or disable the wrap-around effect before starting a game. In the version without the wrap-around effect, a 3-s countdown before the game begins could enhance playability.
- **3D-version:** as suggested in the survey a 3D adaptation (e.g., inspired by *paper.io*(91)) could increase engagement and reduce repetitiveness.
- **Chat and multiplayer:** layers could challenge friends by joining rooms for multiplayer gameplay with chat functionality, making the game more interactive and socially engaging.

### Online Multiplayer Version and Smartphone Optimization

The game could evolve into “*Snakehoot!*”,^92^ an online multiplayer version, inspired by platforms like Kahoot!.^93,94^ Players would compete using preselected diets or element subsets, making it ideal for classroom engagement and formative assessment. *Snakehoot!* could support Formative Assessment Classroom Techniques (FACTs),^93,95^ allowing instructors to assess student understanding in a dynamic and interactive way. Educators could select customizable diets (e.g., elements used in medicine, industry, or space exploration) to structure lesson-based competitions, aligning the game with curriculum objectives. Observations from classroom testing revealed that students naturally engaged in collaborative behaviors during gameplay, often asking classmates or nearby peers for help identifying element symbols or determining whether an element belonged to the selected diet. Some students spontaneously shared frequent mistakes or the names of newly learned elements, fostering peer learning. Additionally, many students challenged themselves and their peers to keep their snake entirely green, avoiding incorrect selections, in addition to aiming for the highest score. These collaborative and competitive tendencies could be enhanced in the multiplayer version. Cooperative game modes could encourage students to work together in team-based challenges, reinforcing discussion and collective problem-solving. Meanwhile, a timed challenge format with leaderboards and ranking systems would boost competitive engagement, motivating students to recall and apply knowledge quickly. To enable this transformation, optimizing the game for smartphones is essential to cater to students’ strong interest in competitive gameplay. Notably, as mentioned in the “Testing” section, one of the main student requests was the ability to challenge one another, reinforcing the demand for a social and interactive learning experience.

### Minor Improvements and Visual Enhancements

These include adding “Back” and “Forward” buttons to allow players to revisit previous choices and a “New Game” button on the game-over screen, enabling a restart with previously selected parameters (e.g., speed, number of elements, and diet) without repeating the setup process (Figure 1). Another enhancement involves the scoring system, introducing a progress bar or a dynamic color indicator that grows with each correct choice, visually representing the player’s accuracy. To enhance fairness and gameplay fluidity, measures will be introduced to prevent elements from appearing consecutively. A formal “win” condition will be implemented, displaying a dedicated message when the snake fills the entire play area, reinforcing a sense of achievement for players who reach this milestone. Additionally, when an element spawns directly in front of the snake (3–5 segments ahead), the game will briefly freeze for 2–3 s, allowing the player time to swap the elements if necessary. A countdown timer may be displayed to indicate the pause duration. To maintain a dynamic and engaging gameplay experience, this freeze time will gradually decrease as the snake grows longer (e.g., down to 1 s in the later stages of the game). Furthermore, graphical enhancements and the integration of engaging soundtracks and audio effects should be implemented to elevate the game’s overall aesthetic appeal^96^ drawing inspiration from games like *paper.io*,^91^*ChemCaper*, *CremCraft*(97) and *Magic Word*.^98^

### Language, Localization, and Global Reach

Translating the game into other languages could greatly enhance its appeal to a global audience. Implementing an access counter and geolocation tracker would allow developers to monitor interest across regions and over time, especially after dissemination events (see Supporting Information).

### Accessibility and Innovative Controls

Using Makey Makey devices^99−101^ or similar technologies, such as Xbox Adaptive Controller,^102^ could enhance accessibility and engagement. These devices allow players to interact with the game through real-world conductive materials, offering a hands-on, multisensory experience. Such simplified interfaces are particularly useful for individuals with both motor and cognitive disabilities.^103^ To further accommodate younger and non-STEM learners, as well as players with accessibility needs, a simplified scoring system will be implemented in this version, displaying only the number of correct choices (see eq 1). This adjustment aims to reduce cognitive load and improve usability without compromising the learning experience. For instance, individuals with cerebral palsy (e.g., infantile stroke, which affects approximately 2 in 1,000 children), autoimmune diseases, spinal cord injuries, or traumatic brain injuries—often caused by road or sports accidents—could greatly benefit from these adaptable controls. By providing an accessible interface, these devices enable diverse audiences, including players with disabilities, to participate more fully and personalize setups for unique learning experiences. In the future, *Snakeleev* aims to collaborate with Emovo Care^104^ to integrate the game with the *Mano* hand exoskeleton,^105^ a wearable robotic device designed to assist and restore hand functions in individuals with motor impairments. Initial evaluations of the *Mano* exoskeleton have demonstrated its ability to restore significant hand functionality in users with spinal cord injuries and to elicit brain activity patterns typical of natural hand movements. By integrating *Snakeleev* with the *Mano* exoskeleton, the goal is to combine sensorimotor training with a ludic and educational activity, creating a synergistic experience that not only aids physical rehabilitation but also fosters cognitive engagement.

### Validating and Refining the Assessment of Effectiveness

The effectiveness findings presented in this study are preliminary and require further validation through controlled research, including direct comparisons between *Snakeleev* and alternative learning methods (e.g., traditional study sessions using element lists for the same duration). Building on previous studies^26,28,106^ future research will establish a structured baseline comparison to more precisely evaluate *Snakeleev*’s educational impact relative to conventional teaching approaches.

### Dissemination and Collaborations

Collaborating with high school textbook publishers, such as Zanichelli and Erickson, could significantly enhance the visibility and adoption of *Snakeleev* in educational environments by including a brief description and a link to the game within their publications. The game will also be showcased at prominent events, including PLAY 2025 - EVOLUTION Festival del gioco in Bologna^107^ and DIDACTA ITALIA 2025 in Firenze.^108^ Additionally, a request has been submitted to feature *Snakeleev* in the 2025 “Il CNR è a scuola” catalog,^109^ which promotes educational initiatives. A potential collaboration with IUPAC^110,111^ could further expand the game’s reach, creating a strong platform for dissemination in both educational and professional contexts. Other initiatives include summer coding schools where high school students refine the game’s code, and structured projects where students and teachers of chemistry and informatics collaborate to align the game with curriculum standards and learning objectives. Furthermore, future efforts will expand the evaluation of *Snakeleev* to diverse educational contexts beyond high school. Studies will involve college-level and non-STEM students to assess the game’s adaptability across different education levels. Alternative thematic diets will also be tested to validate the game’s versatility. Additionally, experimental lessons will be designed around specific diets, integrating short gameplay sessions with traditional teaching methods. For example, a lesson could begin with 5 min of *Snakeleev* gameplay, followed by a lecture, and conclude with another 5 min gameplay session to reinforce learning. Optimized lesson structures will be developed as a guide for educators on effectively integrating *Snakeleev* into classroom instruction and will be made available on platforms such as *LdR*(112) and others.

### Game Expansion and Multidisciplinary Applications: Gamifying Knowledge through the Snake Game

Given the game’s success in terms of enjoyment and effectiveness, a broader vision includes developing themed versions for other disciplines:

- **Snakileo Snakilei:** a physics-focused version of Snake, designed to delve into the world of units of measurement, physical constants, and fundamental principles of physics. Players embark on a journey to collect symbols, values, and equations while mastering topics like energy, force, motion and other key concepts.^113^
- **Phytonacci:** A mathematics version featuring multiplication tables, even and odd numbers, prime numbers, square and triangular numbers, Fibonacci sequences, and more. This version could also include a snake-style Sudoku or challenges involving fractions, multiplication, and other mathematical operations.^114^
- **Snakλíδης:** a geometry-focused version featuring regular polygons, symmetry, and angles.^115^
- **Snakedowska:** a chemistry-themed game honoring Maria Skłodowska Curie.^116^
- **SnEco:** a waste-sorting themed version of Snake that educates players on recycling and sustainability. Players navigate the snake to collect and correctly sort different types of waste—paper, plastic, glass, and organic materials—while learning about their environmental impact and proper disposal methods. Instead of the usual square tiles representing food, this version could feature images of waste items for a more intuitive and visually engaging experiences.^117^
- **Snakonda:** is a visual arts-themed version of *Snake* inspired by *La Gioconda*, immersing players in the world of artistic movements, iconic masterpieces, and creative expression. Players guide the snake to collect and correctly categorize famous artworks, artistic elements such as colors, brushstrokes, and styles, or renowned artists while learning about their historical and cultural significance. Instead of the usual square tiles representing food, this version could feature paintings, sculptures, and digital artworks, creating a visually rich and immersive experience that blends art appreciation with interactive gameplay. To reinforce its artistic identity, the snake’s head could resemble the face from Munch’s *The Scream*, visually embodying the emotional power of art. Players can choose from different diets, each themed around a specific aspect of the visual arts, such as individual artists including Leonardo da Vinci, Van Gogh, and Picasso; artistic periods like Medieval Art, Renaissance, and Baroque; movements such as Impressionism, Cubism, and Surrealism; and types of artworks including oil paintings, sculptures, photography, performance art, land art, and digital art. Additionally, some diets could focus on specific themes found in art, such as serpents. These would feature works like *The Serpent of Asclepius*, a symbol of healing and medicine; *Laocoön and His Sons*; *Medusa* by Caravaggio; *Apollo Killing Python*; *The Fall of Man* by Michelangelo; *Saint George and the Dragon* by Raphael; *Moses and the Bronze Serpent*; *Hercules and the Lernaean Hydra* by Antonio del Pollaiolo; *The Temptation of Saint Anthony* and *The Enigma of William Tell* by Salvador Dalí; and the Aboriginal rock paintings of the *Rainbow Serpent*. The snake’s design could evolve based on the collected artworks: consuming a piece from the “Serpent in Art” diet could grant the snake multiple heads after *Hercules and the Hydra*, a crown after *The Serpent of Asclepius*, glowing eyes after *Medusa*, or a golden aura after *Moses and the Bronze Serpent*. By incorporating historical, mythological, and artistic elements, *Snakonda* transforms art history into an interactive and engaging journey where players discover, categorize, and visualize artistic masterpieces while playing.^118^

The diverse adaptations of Snake presented above—*Snakileo Snakilei* (physics), *Phytonacci* (mathematics), *Snakλíδης* (geometry), *Snakedowska* (chemistry), *SnEco* (waste sorting and sustainability), and *Snakonda* (visual arts)—demonstrate the potential for expanding the *SnakeTheme* concept across various disciplines. In principle, a specific *SnakeTheme* could be developed and implemented in fields such as grammar, literature, music, visual arts, law, and more. Players could select their “snake” (discipline) and “diet” (topic), with adjustable difficulty levels to tailor the educational experience. *Snakehoot!* tournaments could feature combinations of snakes and diets, creating dynamic and challenging gameplay environments. The ultimate goal is to gamify knowledge acquisition by leveraging the simplicity and adaptability of the Snake game, fostering curiosity and active learning across multiple domains.

## Conclusions

This study introduced *Snakeleev*, the first gamified serious game to reimagine the classic *Snake* as an educational tool for learning the periodic table of chemical elements. By integrating active learning, gamification, and real-world applications, *Snakeleev* provides an engaging way for students to recognize and classify chemical elements while fostering interdisciplinary connections.

A preliminary statistical analysis suggested a positive learning effect, with notable score improvements after just 10 min of gameplay, particularly for less familiar topics. Cohen’s d values ranging from 1.23 to 2.67 and *p* < 0.0001 indicate measurable progress, while over 90% of students found the game engaging and helpful. Further studies will explore its effectiveness through comparative analyses and expand its accessibility with multiplayer features, customization, and mobile optimization.

Beyond chemistry education, the flexibility of Snakeleev’s framework paves the way for future applications in other disciplines, such as physics (*Snakileo Snakilei*), mathematics (*Phytonacci*), and sustainability (*SnEco*), underscoring the potential of *Snake*-inspired games as versatile and innovative tools for gamified learning across multiple fields.

## Figures and Tables

**Figure A1:**
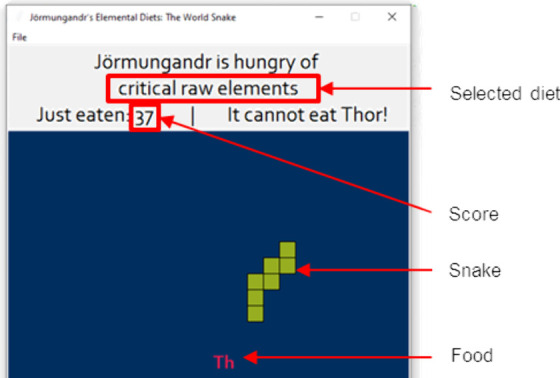
Main window displaying the snake (depicted as segmented green blocks) and the food (represented, in this case, by the Th element). In the header above the game field, the selected diet and current score are highlighted by the red rectangles and pointed to by arrows linked to the corresponding labels. Additionally, the message “It cannot eat Thor!” warns players that consuming the Thor element leads to the end of the game.

## Data Availability

*Snakeleev* code (GitHub repository): https://github.com/PietroGalizia/Snakeleev; *Snakeleev* game (play online): https://pietrogalizia.github.io/Snakeleev/

## Appendix A – Origins of Snakeleev: Blending Mythology, History, and Chemistry

The first version of *Snakeleev* was rooted in Norse mythology, featuring Jörmungandr, the world serpent that encircles Midgard (Earth) by biting its own tail until its final confrontation with Thor during Ragnarök, the apocalyptic battle. This mythological setting was chosen to create an engaging atmosphere and establish a thematic connection with an element from the periodic table: Thorium (Th). In this version, all periodic table elements could be consumed by Jörmungandr, except Thorium. Consuming Thorium resulted in an immediate game over, as explicitly warned in the main window: “*It cannot eat Thor!*” (see Figure A1). This unique mechanic added a layer of strategy while introducing a playful yet educational twist to the conventional rules of traditional snake games. This integration of Norse mythology aimed to inject a sense of fun and originality into the game while providing players with an intuitive yet meaningful way to interact with periodic table concepts. In the view of this author, the portrayal of Thor holds substantial potential for effectively conveying messages to a diverse audience. His choice was further inspired by the widespread cultural resonance of Thor and Jörmungandr. Thor, in particular, has achieved global recognition through his prominent appearances in mainstream media franchises such as the *Avengers* movies, Lego sets, and video games. By leveraging this familiarity, the game was designed to be more appealing and accessible to a broader audience, enhancing its ability to engage players with the educational content in an entertaining and memorable manner.

Similarly, in the Italian version, titled “Serpendeleev: Chimica 3330”, the name pays tribute to Dmitri Mendeleev by combining “serpente” (Italian for snake) and Mendeleev. This wordplay was adapted into *Snakeleev* for the browser-based English version. The subtitle *Chimica 3330* highlights the game’s focus on chemistry while also referencing the Nokia 3330, one of the most iconic mobile phones that popularized the *Snake* game for an entire generation. In this Italian adaptation, the element that triggers a game over is not Thorium but Indium (In) and Tellurium (Te). This change reflects Mendeleev’s approach to classifying elements by atomic mass rather than atomic number, aligning with historical context. Consequently, the warning “It cannot eat Thor!” was replaced with “Attento all’indio e al tellurio!” meaning “Beware of Indium and Tellurium!” This modification retains the game’s educational and playful essence while honoring a unique aspect of Mendeleev’s scientific contributions. Additionally, the Italian chemical names were written in accordance with the latest IUPAC recommendations, ensuring accuracy and consistency with contemporary standards.

## Supporting Information Available

Development history, gameplay evolution, and student survey and assessment results (PDF)
