# Supplementary material for: Snakeleev: A Gamified Serious Game for Learning the Periodic Table
Source: J Chem Educ. 2025 Apr 29;102(5):1814–28. doi: 10.1021/acs.jchemed.5c00029 (PMC12080125; doi:10.1021/acs.jchemed.5c00029)
Supplement: Supplementary file 1 — ed5c00029_si_001.pdf [file ed5c00029_si_001.pdf]

Supporting information

Snakeleev: A Gamified Serious Game for Learning the Periodic Table

Pietro Galizia

National Research Council of Italy - Institute of Science, Technology and Sustainability for Ceramics (CNR - ISSMC), Via Granarolo 64, 48018 Faenza, Italy

E-mail: pietro.galizia@cnr.it

DEVELOPMENT OF SNAKELEEV: FROM PYTHON PACKAGE TO BROWSER GAME

The first version of *Snakeleev* was developed as a Python package, allowing educators and students to explore its functionalities and customize diets or create new levels. The open-source nature of the code encouraged collaborative modifications, fostering an interactive and adaptable learning experience. During its early presentations, including at the Didacta Italia Fair 2024, and through feedback sessions with educators and researchers, two key recommendations emerged: reducing the total number of elements to prioritize commonly encountered ones, and integrating Makey Makey to enhance student engagement with multisensory, hands-on interactions. To overcome compatibility issues with Python installations and to broaden accessibility, the game was re-engineered as a browser-based application using HTML and JavaScript. This transformation ensured seamless gameplay across any device with a browser, significantly improving usability and paving the way for wider adoption in educational settings.

FEEDBACK AND SURVEY RESULTS

1) XXI Convegno Ldr & ChangeGame

During the XXI Conference Ldr on November 21, 2024, in Bologna, a dedicated desk equipped with two PCs allowed high school students to play *Snakeleev*. To attract students to the activity, it was titled “Element Snake” and featured the following description: *"This video game challenges you on the periodic*

table of elements and current topics such as critical materials and sustainability. Your snake is on a diet! Guide the snake to 'eat' only the elements that are part of its diet and avoid the others. Before you know it, you'll learn more about the elements and their uses."

The gameplay session provided an invaluable opportunity to collect student impressions and gain deeper insights into the target audience for the video game. After playing, students were invited to complete a survey, which was answered by four high school students who engaged with the "Elements of a Smartphone" diet. The results, summarized in Table S2, highlighted several key findings. The survey indicated a strong general interest and engagement (Q1, Q2, Q6, Q7, Q8, Q9, and Q16) and a notable effectiveness in fostering learning (Q3, Q4, Q5, and Q10). However, responses were mixed regarding the game's simplicity and clarity (Q3 and Q11). Feedback on design and gameplay was neutral, with challenges cited in the snake's high speed, graphics, and difficulty in recognizing elements relevant to the selected diet (Q11, Q12, Q17, and Q18). It is worth noting that students expressed a positive attitude towards educational games and an interest in the periodic table (Q13, Q14, and Q15). The survey results confirmed the value of Snakeleev as an educational and engaging tool. Players appreciated its innovative approach to teaching the periodic table ("You learn, and it's surprising"), while their constructive feedback provided essential guidance for improvement. To address the identified issues and enhance the user experience, the following changes were implemented:

- Wrap-around effect: preventing the snake from dying when touching the walls.
- Adjustable speed: players can select the snake's speed.
- Enhanced visuals: improved graphics and visual effects to better associate elements with their symbols and identify their relevance to the selected diet.

These refinements led to the version depicted in Figure S1, which differs from the Dual-Element Version described in this article. In this alternative version, players encounter only one element at a time in the game area (Figure S1d), rather than two. To discourage random selection and promote strategic decision-making, a structured scoring system was implemented:

- the score increases when the consumed element belongs to the selected diet;
- the score decreases when the consumed element does not belong to the selected diet.

The scoring system follows these equations, ensuring that random choices do not lead to uncontrolled positive (Eq. S1) or negative (Eq. S2) score divergence:

$$S_{up} = 100\frac{N_{tot} - N}{N_{tot}}; \quad (1)$$

$$S_{down} = 100\frac{N}{N_{tot}}. \quad (2)$$

Where, $S_{up}$and S_{down} represent the positive and negative scores, respectively, N is the number of elements correctly presented in the selected diet, and N_{tot} is the total number of elements selected in the game (see Figure S1b).

Players can press the space bar to discard an element they believe does not belong to the selected diet:

- if the discarded element does belong to the diet, the player loses 1 health point (HP), and the HP bar decreases by one-tenth;
- if the discarded element does not belong to the diet, the player gains 1 HP, and the HP bar increases by one-tenth.

Upon discarding an element, a new one is randomly selected from the periodic table and appears in a random position on the game field.

To enhance visual clarity and learning effectiveness, the HP system will be refined to better distinguish between correct and incorrect choices. Additionally, a highlighted element information feature will be introduced for discarded elements, briefly displaying their name and symbol in an enlarged format—similar to the visualization used for consumed elements (Figure S1d). This addition aims to improve readability and retention without distracting players from controlling the snake.

Furthermore, life-loss mechanics will be adjusted to prevent excessive space bar usage, ensuring a balanced game-over condition across all diets. Notably, in the tested "Elements of a Smartphone" diet, random space bar presses already led to game over, as 82.5% of the 40 selected elements belonged to the chosen diet (Figure S1b).

Table S2. Survey results overview (4 high school students), 21 November 2024. Questions were answered either with YES or NO, on a 5-point Likert scale: strongly disagree (1), disagree (2), neutral (3), agree (4), and strongly agree (5), or open-ended responses. Values are reported as percentages. The questions are numbered and classified into the following categories: interest and engagement (I), learning effectiveness (E), design and gameplay (G), student attitude (A).

#	*Yes/No questions*				*NO*	*YES*
Q1 (I)	Would you play this video game on your smartphone?				0	100
Q2 (I)	Would you recommend this game to someone else (a friend or classmate)?				25	75
Q3 (E)	Were you able to understand which elements are part of the selected diet?				25	50
Q4 (E)	Did you learn any new elements?				0	100
Q5 (E)	Did you learn any new symbols?				25	75
#	*Likert scale questions*	*(1)*	*(2)*	*(3)*	*(4)*	*(5)*
Q6 (I)	I enjoyed playing the game	0	0	0	75	25
Q7 (I)	The game made the classroom activity much more enjoyable	0	0	25	50	25
Q8 (I)	I would play this game outside of school	0	25	50	0	25
Q9 (I)	I would like to play with other diets	0	0	0	50	50
Q10 (E)	I found this game useful for better understanding the periodic table	25	0	25	0	50
Q11 (G)	I found the game simple and easy to play	0	50	50	0	0
Q12 (G)	I liked the design of the game	0	25	50	25	0
Q13 (A)	I think educational games are highly engaging	0	0	0	75	25
Q14 (A)	I am interested in learning the periodic table	0	0	50	25	25
Q15 (A)	I find it fascinating to memorize chemical elements, symbols, and atomic numbers	0	25	0	50	25
#	Open-ended questions					
Q16 (I, G)	What do you like most about this game?<br>Merged replies: the game combines traditional gameplay with modern elements and an educational purpose, offering a surprising and interesting way to learn about elements and their relevance to diets.<br>Highlighted replies: "**You learn, and it's surprising**"					
Q17 (G)	What do you like least about this game?<br>Merged replies: the game's high speed and difficulty make it challenging to control the snake and recognize whether elements belong to the diet. Additionally, dying upon hitting the walls can be frustrating.					
Q18 (G)	What would you improve, change, or add to the game?<br>Merged replies: Reduce the snake's speed, enhance the graphics, and make it clearer and quicker to identify whether elements belong to the selected diet.					

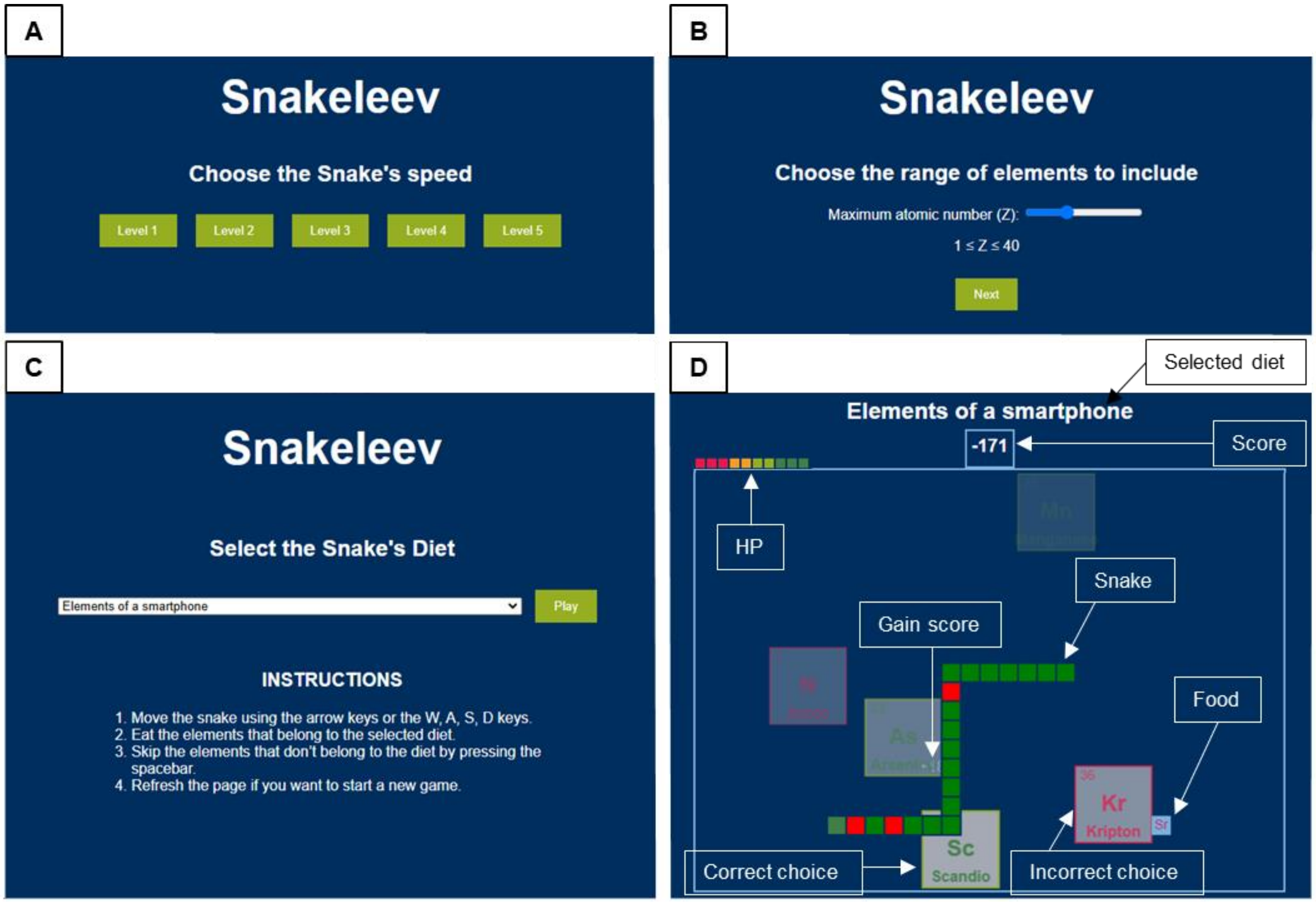

Figure S1. Screenshots of *Snakeleev*'s user interface and gameplay mechanics. (A) The speed selection page allows players to choose the snake's speed, ranging from Level 1 (slowest) to Level 5 (fastest). (B) The element selection page enables players to set the maximum atomic number Z_{max} for the elements appearing in the game, adjustable from 1 to 118. (C) The diet selection and instructions page allow players to choose from 33 thematic *"diets"* of elements, such as *"Elements of a smartphone"*. Each diet highlights different elemental properties or classifications, providing a contextualized learning experience. The page also displays gameplay instructions for controlling the snake and interacting with elements. (D) The gameplay interface highlights key components: the selected diet (top), the score (center), and the health points (HP) bar (top-left). Correctly consumed elements are displayed in green, increasing the score, while incorrectly consumed elements appear in red and decrease the score. The snake grows with each consumed element, and its *"history"* visually records correct (green) and incorrect (red) choices. Players can skip elements by pressing the spacebar, while food (elements) appears randomly on the game field.

2) Istituto Tecnico Tecnologico Statale (ITT) Guglielmo Marconi di Forlì, Italy

At ITT G. Marconi (a technical high school), the class 2E CH, comprising 22 second-year students (equivalent to 10th grade or sophomore year in the U.S. system), participated in the activity. Due to the lack of two consecutive hours, the activity was split into two 50-minute lessons held on December 10 and 13, 2024.

During the first session on December 10, students played the game using the first 30 elements of the periodic table, selecting "Elements of a Smartphone" as the diet. Tests were administered before and after 10 minutes of gameplay to evaluate two skills: associating element symbols with their names (the

A-test) and classifying elements based on the chosen diet (the C-test). These tests are shown in Figure S2a.

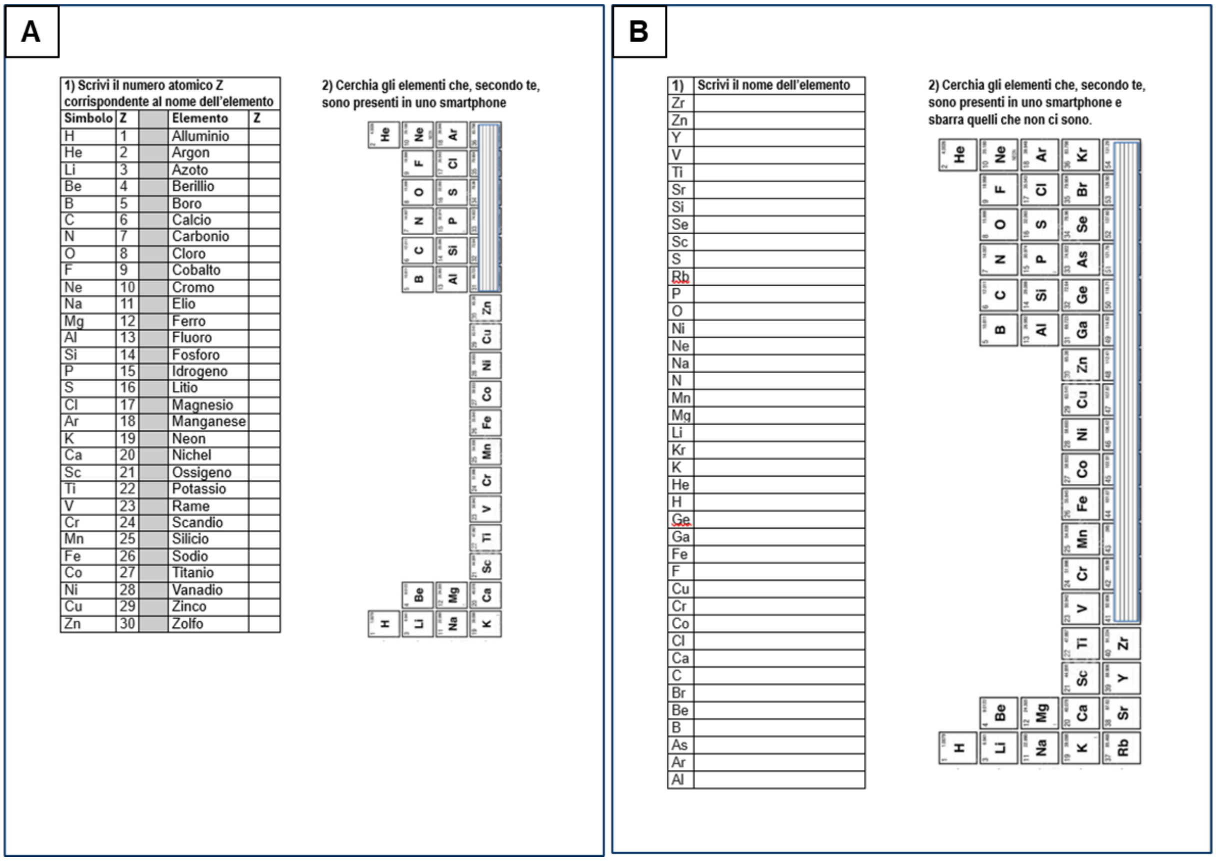

Figure S2 A- and C-tests to assess element-symbol knowledge and classification skills. (a) A-test evaluates the students' ability to associate element symbols with their names, based on a selection of 30 elements (displayed on the left in both panels). (b) C-test evaluates students' ability to classify elements relevant to a specific diet or context, using a selection of 40 elements.

The A-test (Figure S3a,b) proved overly simple despite being time-consuming. Students were not instructed to leave blanks for unknown elements, leading many to adopt strategic approaches. For instance, they first matched symbols uniquely corresponding to names (e.g., "Al" for aluminum) and then used elimination for the rest. This strategy took substantial time and obscured knowledge gaps. Additionally, widespread copying was observed. As a result, the A-test failed to accurately reflect baseline knowledge. Indeed, as shown in Figure S3a, the percentage of correct associations was exceptionally high (97 ± 6%). Notably, sulfur—the most frequent incorrect response in the first test (14%)—was resolved entirely after gameplay (0% incorrect), whereas silicon, initially error-free, rose to 14% incorrect

(Figure S3b). This discrepancy might have been caused by the percentage of students who were copying answers and, due to either errors or intervention by educators, were unable to continue doing so.

In the C-test (Figure S3c,d), students marked elements they believed to be present in a smartphone. The results (Figure S3b) showed a plausible percentage of correct responses (13 ± 6%), supporting its validity. However, students were not instructed to mark elements not in the diet, significantly reducing incorrect responses and limiting assessment of their classification skills. Nonetheless, gameplay helped students recognize the diversity of elements in a smartphone, revealing that far more are involved than initially assumed. For example, lithium (essential for batteries) and cobalt (linked to social and environmental issues in coltan mining) were initially overlooked but more frequently identified in the second test. Similarly, elements critical to plastic components, like carbon and hydrogen, were excluded initially despite their obvious presence. Another significant oversight was silicon—essential for electronics and glass—which many failed to recognize. The omission of oxygen was also striking, considering its critical role in materials like ceramics used in capacitors and insulators. These gaps underscored opportunities to teach foundational materials science concepts, emphasizing oxygen and other key elements in modern technology.

Beyond the scientific aspects, the exercise prompted broader discussions on the economic, environmental, and geopolitical implications of material use. Elements like lithium and cobalt are not only essential for functionality but also central to debates on ethics and sustainability. Their extraction often involves significant environmental degradation and labor exploitation, particularly in regions with coltan mining. These discussions highlighted the interconnectedness of materials science with pressing global issues, encouraging students to think critically about the lifecycle of technological devices, the importance of recycling, and the broader implications of their use. Through these reflections, the C-test and game demonstrated their effectiveness as tools for integrating technical knowledge with social awareness.

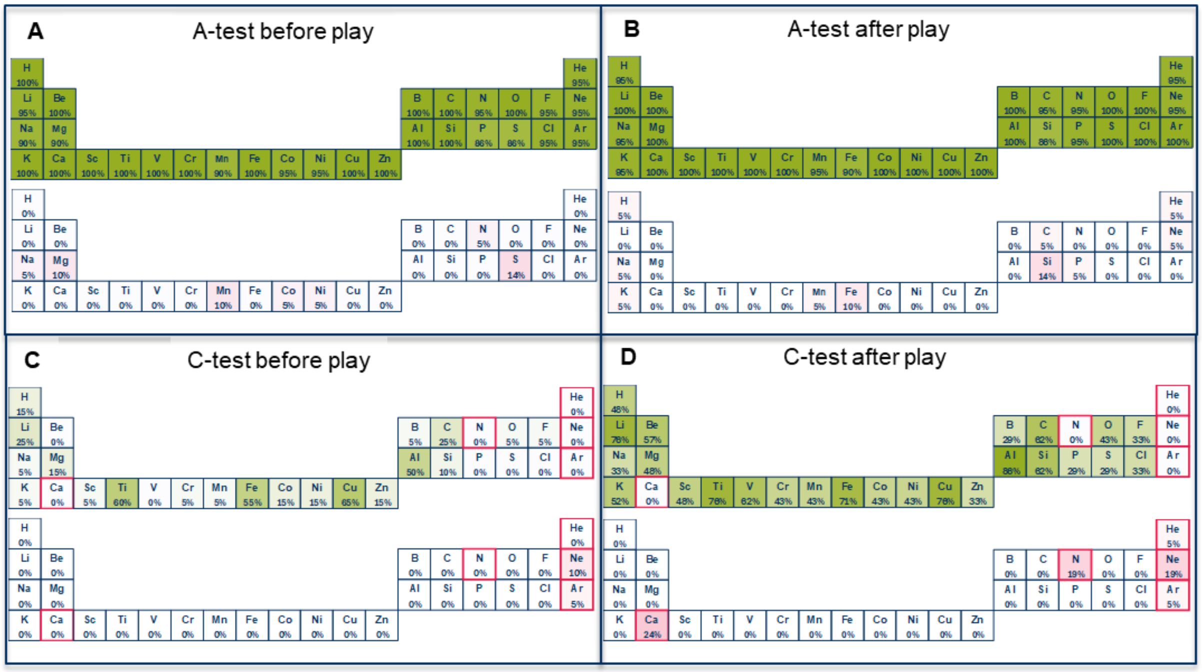

Figure S3 Heat maps of the periodic table illustrating student performance in (a) the A-test: association of element symbols with their corresponding names, and (c) the C-test: classification of elements as part of the selected diet ("Elements of a Smartphone") before playing Snakeleev. Green shading indicates the percentage of correct responses, while red shading highlights the percentage of incorrect responses. Elements with red-outlined borders in the C-test represent those not included in the selected diet. The heat maps for (b) the A-test and (d) the C-test show student performance after playing Snakeleev for 10 minutes.

To address test-related limitations, both assessments were revised (Figure S2b). The number of elements was increased from 30 to 40, and the order of elements in the A-test was randomized across sheets. For the C-test, students were instructed to circle elements present in a smartphone, cross out those not present, and leave unknown elements unmarked. The updated tests (Figure S2b) were administered on December 13. Despite these improvements, the same groups of students continued to copy from one another, aiming to perform well. Many avoided leaving blank spaces, instead inventing names for unknown elements (e.g., writing "YUPSIA" for yttrium and "VANESIO" for vanadium). During gameplay, some students opted for maximum snake speed, despite advice to use intermediate speed (level 3) to balance enjoyment with sufficient time for processing element names and symbols.

Examining the A-test results (Figure S4a,b), students initially displayed high accuracy for familiar elements like hydrogen, lithium, and helium (all 100%). This reflects their foundational chemistry

knowledge. Conversely, less familiar elements, such as yttrium and zirconium, had higher percentages of missing responses, highlighting significant gaps. Incorrect responses were rare initially, as students often left questions unanswered when unsure. After gameplay, performance improved notably for elements with lower initial accuracy. For instance, correct responses for yttrium and zirconium increased significantly, showing the game's effectiveness in reinforcing knowledge. Missing responses decreased substantially, indicating greater confidence post-intervention. However, some elements, like calcium, showed slight increases in incorrect responses, possibly due to gameplay-induced confusion or overconfidence. Precision analysis revealed further insights. Familiar elements like oxygen and nitrogen consistently demonstrated near-perfect precision, reflecting robust foundational knowledge. In contrast, less familiar elements, such as zirconium and gallium, remained less precise even after gameplay. This suggests that while students could associate these elements with their symbols, their understanding remained superficial after 10 minutes of play. Overall, the game significantly enhanced students' knowledge of element symbols and associations, particularly for less familiar elements. The marked reduction in missing responses highlights increased engagement and confidence post-intervention. However, elements like gallium and arsenic still showed modest accuracy and precision, indicating a need for further emphasis. Suggestions, such as providing tailored advice on the game-over page ("Stay hungry, stay periodic!" page), could help address these gaps. It is worth noting that this feature was not activated in the version of Snakeleev used during testing.

These findings underline the potential of educational games to reinforce foundational knowledge while identifying areas for pedagogical development. High performance on familiar elements prior to gameplay suggests the A-test may benefit from including more challenging elements to better differentiate knowledge levels. Repeated gameplay also proved effective for consolidating classification skills in the C-test. It's important to note that the students had already engaged with the same game and dietary theme just three days earlier, which likely influenced the results and a degree of retention from the previous gameplay was evident. Correct responses showed slight improvements across several elements, reflecting an incremental consolidation of knowledge. However, there were still notable missing responses, particularly for elements less emphasized in typical chemistry curricula, such as gallium, arsenic, and selenium. Incorrect responses were slightly more frequent than before, possibly due to

lingering confusion about the classification of certain elements. After playing again on December 13th, the results showed a more substantial improvement in correct responses across the board. Elements like magnesium, calcium, and zinc were increasingly recognized. Additionally, the precision of correct answers improved significantly, indicating a deeper understanding of the material. The reduction in missing responses suggests increased confidence among students, while incorrect answers decreased for most elements, pointing to the effectiveness of the game in clarifying misconceptions. Overall, these results highlight the value of repeated exposure to the game in enhancing students' classification skills.

The results presented in Figure S5 further reinforce the educational potential of Snakeleev. For the A-test (Figure S5a), while correct responses were already high prior to gameplay, due to the simplicity of the first test (Figure S2a), precision improved notably post-intervention, reflecting the game's effectiveness in reinforcing students' association skills. Missing responses were significantly reduced, suggesting increased confidence in their knowledge. The modified A-test on December 13 (Figure S2b) led to a clear rise in correct responses and a corresponding decrease in incorrect ones, demonstrating that the revised format improved the test's ability to assess knowledge gaps and enhance learning outcomes. For the C-test (Figure S5b), the improvement in correct classifications, even after a three-day pause, indicates that the game successfully reinforced students' understanding of elements linked to the selected diet ("Elements of a Smartphone"). The broader range in precision rates reflects individual variability in grasping the content, suggesting that while Snakeleev effectively consolidates foundational knowledge, there remains an opportunity to address specific learning gaps.

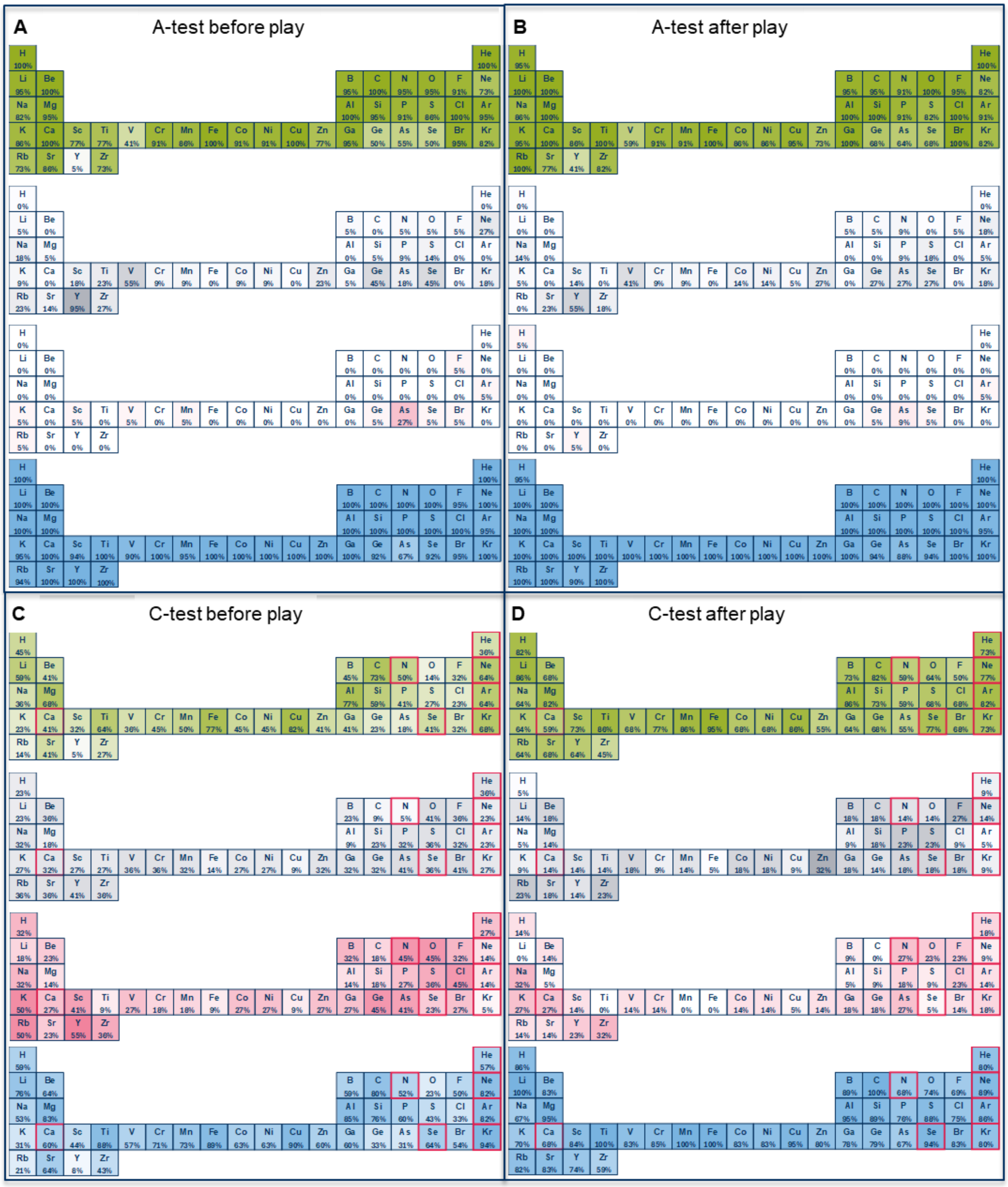

210 Figure S4 Heat maps of the periodic table illustrating student performance in (a) the A-test: association of element symbols with their corresponding names, and (c) the C-test: classification of elements as part of the selected diet ("Elements of a Smartphone") before playing Snakeleev. Green shading indicates the percentage of correct responses, gray shading represents the percentage of missing responses, red shading highlights the percentage of incorrect responses, and blue shading highlights the precision of the correct responses. Elements with red-outlined borders in the C-test represent those not included in the selected diet. The heat maps for (b) the A-test and (d) the C-test show student performance after playing Snakeleev for 10 minutes.

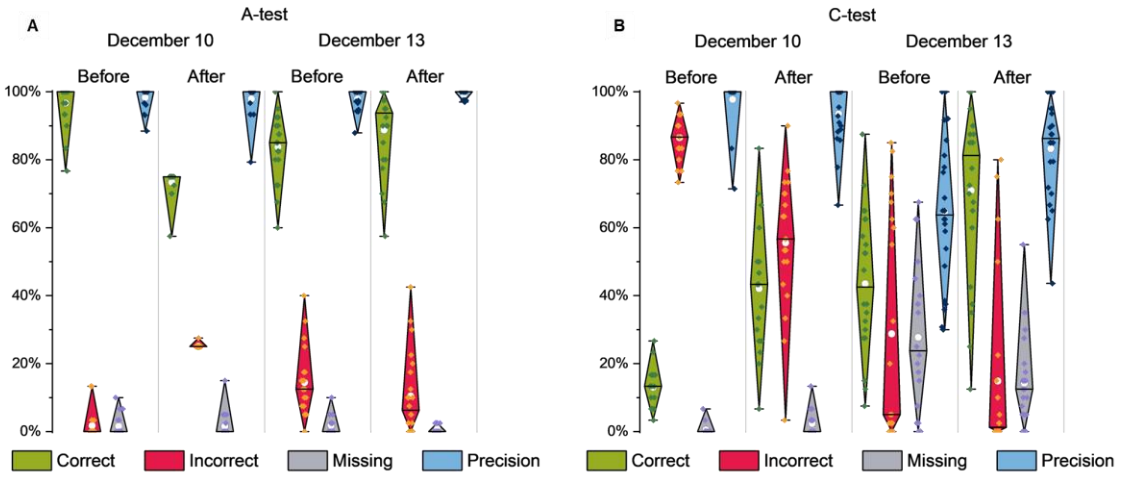

Figure S5 Performance of 22 students in the A-test and C-test before and after playing Snakeleev for 10 minutes. (a) The A-test evaluated students' ability to associate element symbols with their corresponding names, while (b) the C-test assessed their ability to identify elements linked to the "Elements of a Smartphone" diet. Correct responses are shown in green, incorrect responses in red, missing responses in gray, and precision rates in blue. The diamond-shaped boxes represent the full range of percentages from the lowest to the highest scores among students, with the horizontal line (minor diagonal) dividing the responses into two equal groups. The white spot within each box indicates the mean percentage of the responses.

3) Liceo Scientifico Torricelli-Ballardini di Faenza, Italy

At the Torricelli-Ballardini high school, two second-year classes (equivalent to 10th grade or sophomore year) participated in the study: Class 2ES (27 students, December 16, 2024) and Class 2FS (24 students, December 17, 2024). Unlike the previous tests conducted on December 13, the methodology was slightly adjusted. The maximum speeds (levels 4 and 5) were slowed from 100 ms and 50 ms per movement to 125 ms and 100 ms, respectively. Instead of presenting Tests A and C side by side on the same sheet (Figure S2b), each test was displayed on separate pages to enhance focus. Additionally, time management was more strictly controlled, although some students found ways to influence each other and copy responses. For both classes, the process began with distributing the Test-A sheets face down. Students were instructed to turn the sheets over and complete Test A within 4 minutes. Next, they flipped to Test C and had 2 minutes to complete it. After 10 minutes of playing Snakeleev, they repeated the tests under the same conditions. At the end, students completed a survey, allowing additional time for feedback and discussion about the game. This included insightful remarks

such as, *“Prof, this Snake is better than that of Google!”*. Furthermore, within the 100 minutes taken for test Snakeleev, brief lessons on criticality of elements, electronic device composition, and principles of circular economy and sustainability were integrated into the session.

Results for Class 2ES: the survey of 27 high school students (Table S3) confirmed strong general interest and engagement with Snakeleev (Q1, Q2, Q6, Q7, and Q16). A high percentage (85%) expressed willingness to play the game on their smartphones, and 96% would recommend it to others. While students found the gameplay enjoyable and productive, most (70%) indicated they would use it only as an educational tool, not for leisure (Q8 and Q9).

In terms of learning effectiveness, the game proved highly impactful (Q3), with 89% of participants reporting they learned new elements (Q4) and 93% stating they learned new symbols (Q5). On average, students reported memorizing 2.4 elements after playing for 20 minutes (Figure S6). Furthermore, 85% of respondents found the game helpful in enhancing their understanding of the periodic table (Q10).

The design and gameplay received mixed feedback (Q11 and Q12). While 77% found the game simple to play, students pointed out areas for improvement, particularly regarding graphics, responsiveness, and clarity. Suggestions included adding features such as leaderboards, multiplayer modes, and enhanced visuals (Q17 and Q18).

As for student attitudes, 74% found educational games engaging (Q13), and a notable portion (52%) expressed interest in learning the periodic table (Q14). The survey indicates that Snakeleev is an engaging and effective educational tool, successfully blending gameplay and learning. However, addressing feedback on design, user interface, and additional features could enhance its long-term appeal.

Table S3. Survey results overview (27 high school students), 16 December 2024. Questions were answered either with YES or NO, on a 5-point Likert scale: strongly disagree (1), disagree (2), neutral (3), agree (4), and strongly agree (5), or open-ended responses. Values are reported as percentages. The questions are numbered and classified into the following categories: interest and engagement (I), learning effectiveness (E), design and gameplay (G), student attitude (A).

#	*Yes/No questions*				*NO*	*YES*
Q1 (I)	Would you play this video game on your smartphone?				7	85
Q2 (I)	Would you recommend this game to someone else (a friend or classmate)?				4	96
Q3 (E)	Were you able to understand which elements are part of the selected diet?				7	89
Q4 (E)	Did you learn any new elements?				11	89
Q5 (E)	Did you learn any new symbols?				7	93
#	*Likert scale questions*	*(1)*	*(2)*	*(3)*	*(4)*	*(5)*
Q6 (I)	I enjoyed playing the game	0	0	15	67	18
Q7 (I)	The game made the classroom activity much more enjoyable	0	0	7	37	56
Q8 (I)	I would play this game outside of school	0	37	33	26	4
Q9 (I)	I would like to play with other diets	0	7	22	63	8
Q10 (E)	I found this game useful for better understanding the periodic table	0	4	11	48	37
Q11 (G)	I found the game simple and easy to play	0	4	19	44	33
Q12 (G)	I liked the design of the game	4	41	29	22	4
Q13 (A)	I think educational games are highly engaging	0	3	41	41	15
Q14 (A)	I am interested in learning the periodic table	0	15	52	33	0
Q15 (A)	I find it fascinating to memorize chemical elements, symbols, and atomic numbers	7	15	37	33	8

#	Open-ended questions
Q16 (I, G)	What do you like most about this game?<br>Merged replies: students appreciated the game's ability to teach the periodic table in a simple, enjoyable, and alternative way. Many highlighted how the game helps with memorization while relaxing or playing, combining fun and learning. Others emphasized its practical and effective design, which makes understanding elements and classifications easier.<br>Highlighted replies:<br>• *"**Even without trying, you automatically learn the periodic table quickly.**"*<br>• *"Its simplicity and effectiveness."*<br>• *"**It definitely saves me hours of studying.**"*<br>• *"It's cute and, at the same time, helps memorize elements and their classifications."*<br>• *"It provides an alternative way to learn elements."*<br>• *"**It helps you memorize elements while relaxing.**"*
Q17 (G)	What do you like least about this game?<br>Merged replies: students mentioned difficulties such as the snake's design (a "block") and frustrations with controls and input responsiveness. Other complaints included the lack of visual feedback when pressing the spacebar, repetitive gameplay, and the simple graphics. Suggestions included a smoother start to gameplay and better visual clarity.
Q18 (G)	What would you improve, change, or add to the game?<br>Merged replies: suggestions focused on enhancing competitiveness and interactivity, such as adding leaderboards, multiplayer options, and personal records. Visual improvements included more detailed graphics, a customizable snake, and grid enhancements. Functional suggestions included a pause button, better feedback on correct/incorrect inputs, and expanded game modes with additional features such as music and home-screen navigation.

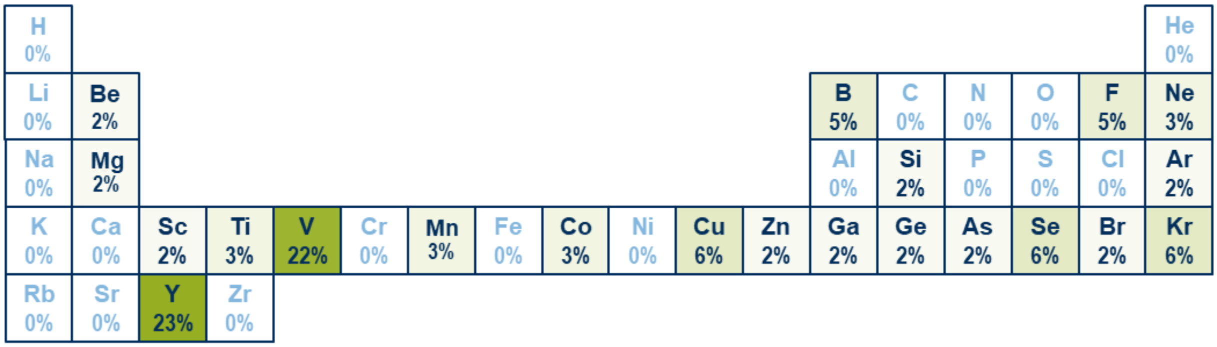

Figure S6 Distribution of elements declared to have been memorized after 20 minutes of playing Snakeleev. Each player could declare a maximum of 5 elements. Out of 120 possible elements, 68 were reported in the survey, with an average of 2.8 elements per student.

Results for Class 2FS: Compared to the previous class, where the main feedback emphasized the desire to play together and retain scores, this class strongly highlighted the need for a pause button. This requirement became particularly apparent when, after 10 minutes of gameplay, students were asked to stop and perform the A-test. Many students, having achieved long, error-free snakes (indicated by the entirely green snake), were reluctant to abandon their progress. Additionally, this survey revealed that gameplay satisfaction was impacted by the limited number of “incorrect” elements in the selected diet (only 17.5% of the first 40 elements). While this reduced the game’s challenge, it effectively emphasized the educational point about the amount of elements present in a smartphones. Similarly, the scoring system—designed to oscillate around zero when elements were chosen at random—drew criticism for heavily penalizing incorrect selections. A particularly interesting suggestion was to make the game 3D, while others compared *Snakeleev* to *Paper.io*. This comparison opens up potential future developments, such as improving graphics and integrating features inspired by *Paper.io*. In an informal survey, I also asked students whether they were familiar with the classic *Snake* game and if they had played it. Out of 19 students who answered, 18 knew the game, and 8 out of 11 had played it. This finding suggests that *Snake* remains widely recognized, providing further motivation to continue developing *Snakeleev*.

The broader survey reinforced that the educational video game was generally well-received (Q1, Q2). Students appreciated its ability to make learning the periodic table enjoyable and effective (Q3, Q4, Q5, Q6, Q7, Q10), though fewer expressed a strong interest in playing it outside of school (Q8). However,

many liked the idea of exploring other diets within the game (Q9). Notably, students have reported to had averagely learn 2.4 elements (see Figure S7).

Regarding gameplay, the design and mechanics received mixed feedback. While some found the game easy to play (Q11), others noted issues with visibility, speed, and graphics (Q17, Q18). The game's creative and educational premise, along with its engaging gameplay, was widely praised for helping players memorize elements in a fun and lightweight manner. Nonetheless, students identified areas for improvement, such as adding a pause button, enhancing the graphics, and refining mechanics to reduce repetitiveness and improve usability.

Overall, the feedback underscores the game's strong potential as an educational tool while emphasizing the need for specific adjustments to enhance the user experience and accessibility.

Table S4. Survey results overview (24 high school students), 17 December 2024. Questions were answered either with YES or NO, on a 5-point Likert scale: strongly disagree (1), disagree (2), neutral (3), agree (4), and strongly agree (5), or open-ended responses. Values are reported as percentages. The questions are numbered and classified into the following categories: interest and engagement (I), learning effectiveness (E), design and gameplay (G), student attitude (A).

#	*Yes/No questions*				*NO*	*YES*
Q1 (I)	Would you play this video game on your smartphone?				21	79
Q2 (I)	Would you recommend this game to someone else (a friend or classmate)?				8	88
Q3 (E)	Were you able to understand which elements are part of the selected diet?				4	92
Q4 (E)	Did you learn any new elements?				0	100
Q5 (E)	Did you learn any new symbols?				8	92
#	*Likert scale questions*	*(1)*	*(2)*	*(3)*	*(4)*	*(5)*
Q6 (I)	I enjoyed playing the game	0	0	8	79	13
Q7 (I)	The game made the classroom activity much more enjoyable	0	0	4	33	63
Q8 (I)	I would play this game outside of school	8	17	46	25	4
Q9 (I)	I would like to play with other diets	0	4	21	67	8
Q10 (E)	I found this game useful for better understanding the periodic table	0	4	21	33	42
Q11 (G)	I found the game simple and easy to play	0	4	17	50	29
Q12 (G)	I liked the design of the game	17	21	42	17	4
Q13 (A)	I think educational games are highly engaging	4	0	29	42	25
Q14 (A)	I am interested in learning the periodic table	4	0	42	50	4
Q15 (A)	I find it fascinating to memorize chemical elements, symbols, and atomic numbers	17	8	17	29	29
#	Open-ended questions					
Q16 (I, G)	What do you like most about this game?<br>Merged replies: students appreciated the game for its simplicity, creativity, and ability to help them learn and memorize chemical elements in a fun and engaging way. They particularly liked how the game combines mental challenges, visual memory, and educational content, making it an enjoyable pastime that teaches even lesser-known elements.<br>Highlighted replies:<br>• *“While playing, visual memory helps me focus and learn.”*<br>• *“The simplicity and ability to help you memorize elements.”*<br>• *“You learn the names of elements and where they are used in a fun way.”*<br>• *“Learning while playing.”*<br>• ***“It’s a good pastime, and it helps you learn.”***<br>• ***“It allows you to memorize elements in a light-hearted way.”***<br>• ***“I learn while having fun.”***					
Q17 (G)	What do you like least about this game?<br>Merged replies: the main issues students raised were the lack of a pause button, repetitive gameplay, and limited visibility of certain elements on the screen. Some also mentioned frustration with the graphic design and the inability to view their final score after the game ends.					
Q18 (G)	What would you improve, change, or add to the game?<br>Merged replies: Students suggested adding a pause button, improving the graphics (e.g., smoother shapes, 3D options, customizable snake colors), and increasing the visibility of elements on the screen. They also recommended features like keeping track of the best score in the menu, allowing the game to resume without reselecting options, and diversifying the elements shown during gameplay.					

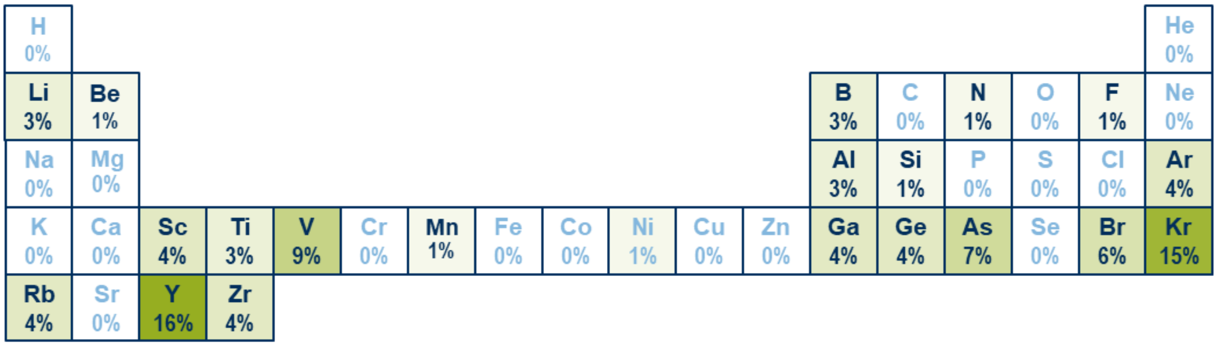

Figure S7 Distribution of elements declared to have been memorized after 20 minutes of playing Snakeleev. Each player could declare a maximum of 5 elements. Out of 120 possible elements, 68 were reported in the survey, with an average of 2.8 elements per student.

The results on learning effectiveness tests depicted in Figure S8 illustrate the impact of playing Snakeleev on students' ability to associate element symbols with their corresponding names (A-test). Prior to gameplay, the correct response rates (Figure S8a) were generally high for well-known elements, reflecting foundational knowledge. However, less familiar elements, such as yttrium and zirconium, exhibited lower accuracy, underscoring gaps in students' understanding. After 10 minutes of gameplay, significant improvements in correct responses were observed, particularly for previously challenging elements, demonstrating the game's effectiveness in reinforcing symbolic knowledge. This trend continued after an additional session of gameplay, with further reductions in missing responses (Figure S8c), indicating increased confidence and engagement.

Despite this progress, some elements, such as gallium and arsenic, showed persistent inaccuracies, highlighting the need for additional instructional scaffolding or targeted gameplay interventions. Notably, errors with arsenic often stemmed from the association of its symbol (As) with argon (Ar) or the intuitive yet incorrect association with astatine (At). The precision of responses (Figure S8d) also improved markedly post-intervention, particularly for less familiar elements, further emphasizing the educational potential of Snakeleev. However, the slight increase in incorrect responses for certain elements, such as calcium, may suggest a trade-off between enhanced engagement and the potential for overconfidence or misclassification during gameplay. Overall, the figure underscores the game's ability to enhance knowledge retention and confidence in associating element symbols with their names, while also identifying areas where additional pedagogical support could further optimize learning outcomes.

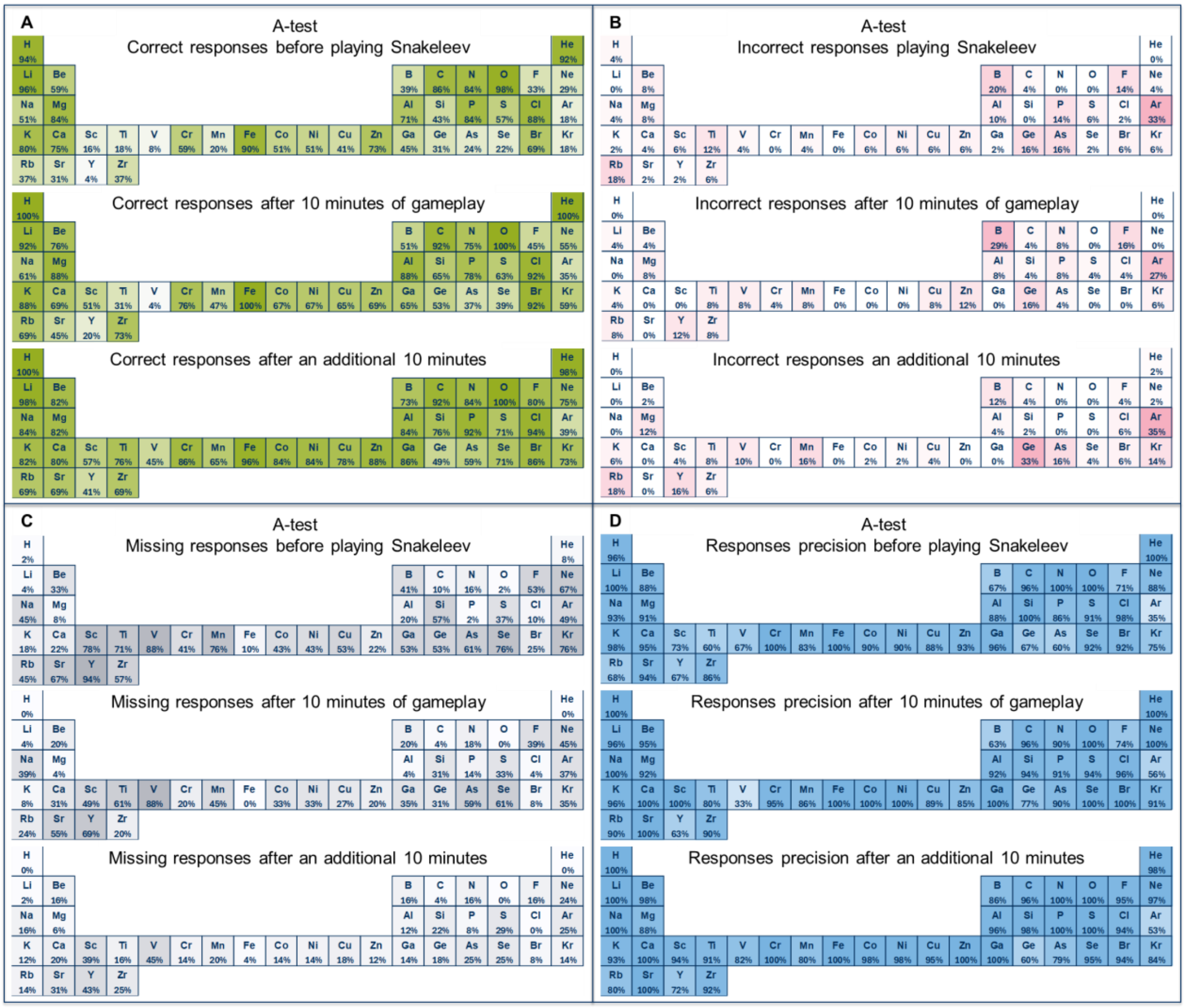

Figure S8 Heat maps of the periodic table showing student performance in the A-test (associating element symbols with their names) before playing Snakeleev, after 10 minutes of gameplay, and after an additional 10 minutes. (a) Green shading indicates the percentage of correct responses, (b) red shading shows the percentage of incorrect responses, (c) gray shading represents missing responses, and (d) blue shading highlights the precision of correct responses.

The results of the C-test shown in Figure S9 reveal the impact of playing Snakeleev on students' ability to classify elements related to the "Elements of a Smartphone" diet. Before gameplay, the accuracy of correct classifications (Figure S9a) was modest, particularly for elements less directly associated with the selected context, such as scandium and yttrium. After the first 10-minute session, improvements in correct responses were observed, with notable gains for elements like gallium and indium, which are critical to the diet theme. These improvements continued following an additional session of gameplay, as evidenced by the reduction in missing responses (Figure S9c), suggesting that Snakeleev fosters deeper engagement and retention. The precision rates (Figure S9d) also improved, demonstrating an

overall increase in students' ability to accurately classify elements. However, the variability in incorrect responses (Figure S9b) for some elements hints at individual differences in prior knowledge or potential overgeneralization during gameplay. These findings highlight Snakeleev's potential to enhance thematic understanding in a gamified context while pointing to areas where additional support or clarification could optimize learning outcomes.

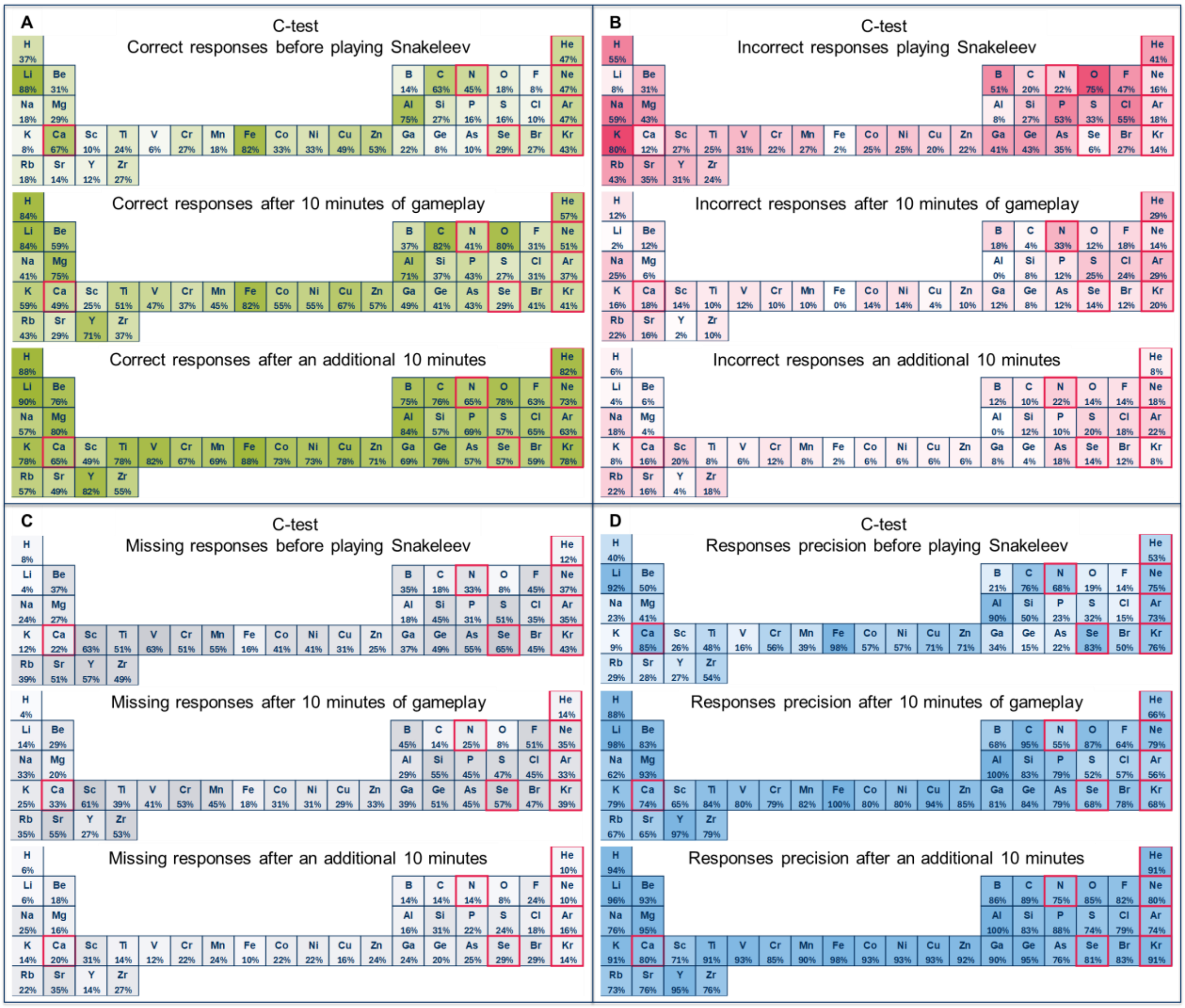

Figure S9 Heat maps of the periodic table depicting student performance in the C-test (classifying elements as part of the selected diet: 'Elements of a Smartphone') before playing Snakeleev, after 10 minutes of gameplay, and after an additional 10 minutes. (a) Green shading represents the percentage of correct responses, (b) red shading shows incorrect responses, (c) gray shading indicates missing responses, and (d) blue shading highlights the precision of correct responses. Red-outlined elements represent those not included in the selected diet.

The results presented in Figure S10 and Table S5 confirm and extend the trends observed in Figures S8 and S9, highlighting the impact of Snakeleev on students' ability to associate element symbols with

their names (A-test) and classify elements related to the "Elements of a Smartphone" diet (C-test). Before gameplay, performance in both tests showed significant variability. In the A-test, correct response rates reflected foundational knowledge, while the C-test exhibited only modest initial accuracy. After 10 minutes of gameplay, however, both tests demonstrated substantial improvements in correct responses (+13% for A-test and +20% for C-test), along with a marked reduction in incorrect and missing answers. The upward trend continued after an additional 10 minutes of gameplay. Correct responses reached 78% in the A-test and 70% in the C-test, underscoring Snakeleev's effectiveness. By integrating the results of Figure S10a with those of Figure S8, it becomes evident that the A-test saw significant gains in correct responses for less familiar elements, illustrating how gameplay helped address gaps in students' understanding of elemental symbols. Similarly, the C-test results, when considered alongside Figure S9, reveal notable improvements in the classification of elements like gallium and indium. The sustained increase in precision rates across both tests underscores Snakeleev's potential to foster deeper comprehension and confidence in a relatively short period of time.

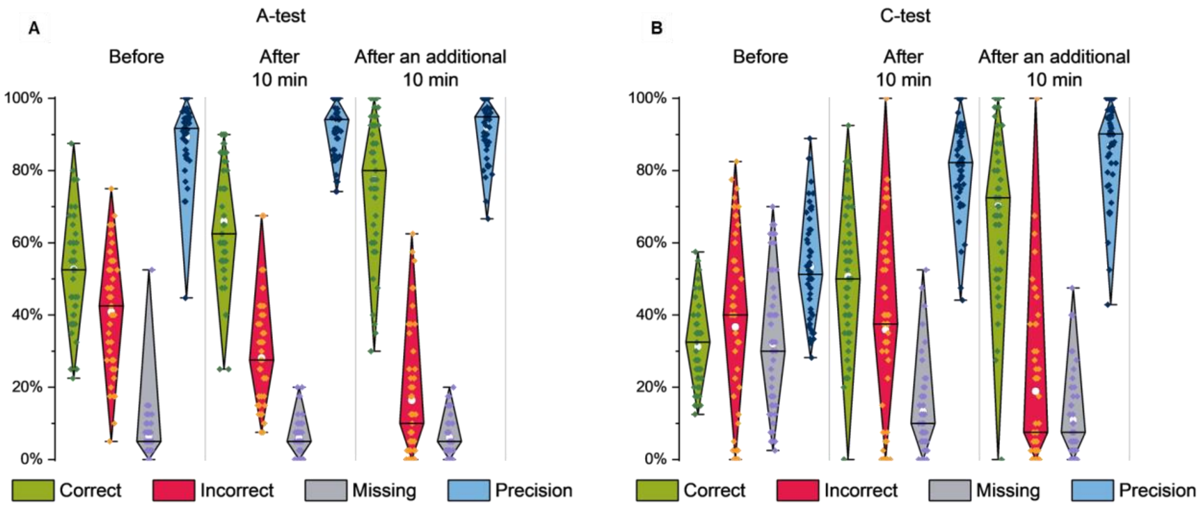

Figure S10 Performance of 51 students in the A-test and C-test before playing Snakeleev, after 10 minutes of gameplay, and after an additional 10 minutes. (a) The A-test evaluated students' ability to associate element symbols with their corresponding names, while (b) the C-test assessed their ability to identify elements linked to the "Elements of a Smartphone" diet. Correct responses are shown in green, incorrect responses in red, missing responses in gray, and precision rates in blue. The diamond-shaped boxes represent the full range of percentages from the lowest to the highest scores among students, with the horizontal line (minor diagonal) dividing the responses into two equal groups. The white spot within each box indicates the mean percentage of the responses.

Table S5. Descriptive statistics summarizing the performance of 51 students in the A-test and C-test before playing Snakeleev (I), after 10 minutes of gameplay (II), and after an additional 10 minutes (III). For each test, the table presents the mean values (percentages for responses and scores), standard error of the mean (±), standard deviation (σ) and p-values for the Shapiro-Wilk (p) tests for normality. Correct responses, incorrect responses, missing responses, and calculated scores are included.

	A-test				*C-test*			
Responses	*Mean*	±	*σ*	*p*[a]	*Mean*	±	*σ*	*p*[a]
Correct								
I	53%	2%	16%	0.30	31%	2%	11%	0.11
II	66%	2%	17%	0.01	51%	3%	19%	0.92
III	78%	3%	19%	>0.01	70%	3%	22%	0.02
Incorrect								
I	41%	2%	17%	0.54	37%	4%	27%	>0.01
II	28%	2%	15%	0.01	36%	3%	26%	0.01
III	16%	2%	17%	>0.01	19%	3%	22%	>0.01
Missing								
I	6%	1%	8%	>0.01	32%	3%	19%	0.01
II	6%	1%	5%	>0.01	13%	2%	12%	>0.01
III	6%	1%	6%	>0.01	11%	2%	12%	>0.01
Score[b]								
I	18.5	1.0	7.3	0.54	-0.3	1.0	6.8	0.01
II	24.0	1.1	7.8	0.02	15.0	1.1	7.7	0.76
III	28.5	1.2	8.9	0.01	23.7	1.5	10.7	0.18

[a]At the 5% significance level, normality is not rejected if $p > 0.05$, while normality is rejected if $p < 0.05$.
[b] Scores were calculated individually for each of the 51 students by assigning +1 for each correct response and −1 for each incorrect response.
